# Supplementary material for: Systematic functional analysis of rab GTPases reveals limits of neuronal robustness to environmental challenges in flies
Source: eLife. 2021 Mar 5;10:e59594. doi: 10.7554/eLife.59594 (PMC8016483; doi:10.7554/eLife.59594)
Supplement: Supplementary file 1. — (A) Expression notes on optic lobe expression at 40% pupal development. (B) Expression notes on adult brains. The expression patterns are shown in Figure 1—figure supplements 2–3. [file elife-59594-supp1.docx]

**Supplementary Table / Supplementary File 1**

**A: Expression in the developing optic lobe (P+40%)**

| **EYFP-Rab** | **Expression notes** |
| --- | --- |
| **Rab3** | Strongly enriched in the synaptic neuropils medulla, lobula and lobula plate. Weakly expressed in the lamina. Weak punctate labeling in cellular cortex regions. No discernable expression in the retina. |
| **RabX4** | Intermediate levels in both neuropils and cellular cortex regions. Mildly enriched in the lamina. Weak expression in the retina. |
| **Rab27** | Very weak expression at this developmental stage. |
| **Rab26** | Strongly enriched in the synaptic neuropils medulla, lobula and lobula plate. Weakly expressed in the lamina. Weak punctate labeling in cellular cortex regions. Weak expression in the retina. |
| **Rab19** | Strongly expressed in the lamina and specific regions of the medulla, lobula and lobula complex. Strongly expressed in the cell cortex region adjacent to the lobula plate, weaker in all other cellular cortex regions. Intermediate expression in the retina. |
| **Rab32** | Strongly expressed only in photoreceptor neurons, including cell bodies in the retina, axons and terminals in the lamina and medulla. |
| **RabX1** | Strongly expressed in all cellular cortex regions; largely excluded from synaptic neuropils. Weak expression in the retina. |
| **RabX6** | Intermediate enrichment in all synaptic neuropils and relatively weaker expression in cellular cortices. Intermediate to strong expression in the retina. |
| **Rab40** | Weak expression throughout all synaptic neuropils, cellular cortices and in the retina. |
| **Rab23** | Strongly but sparsely expressed in a few visual neurons, around the lobula, and in the retina, here especially bristle cells. |
| **Rab21** | Weakly expressed in both synaptic neuropils and cellular cortices. Slightly enriched in the lamina and retina. |
| **Rab9** | Weakly expressed in all cellular cortices with mild enrichment in the outer layer of the brain, the synaptic neuropils and the retina. |
| **Rab4** | Strongly expressed in all cell cortices with a particular enrichment in cells near the lobula plate. Mild enrichment in the retina and in specific neuropil layers, including the distal medulla and the lamina. |

**B: Adult neuropil expression pattern**

| **EYFP-Rab** | **Expression notes** |
| --- | --- |
| **Rab3** | Strongly expressed in most or all synaptic neuropils, with particular enrichment in the mushroom body, ellipsoid body and other central brain regions. Relatively weaker expression in the lamina. Largely excluded from cell bodies. |
| **RabX4** | Strongly and relatively evenly expressed in most or all synaptic neuropils. Relatively weaker expression in the lamina. Weak expression in cell bodies. |
| **Rab27** | Strongly expressed in specific medulla layers, mushroom bodies and some central synaptic neuropils. Relatively weaker expression in many other synaptic neuropil regions, including the lamina and lobula complex. Largely excluded from cell bodies. |
| **Rab26** | Strongly expressed in specific synaptic neuropil regions, including distal and proximal medulla layers, the outer central domain of the ellipsoid body (EBoc), the posterior lateral protocerebrum, and the calyx of the mushroom body. Relatively weaker expression in the lamina. Largely excluded from cell bodies. |
| **Rab19** | Strongly and relatively evenly expressed in synaptic neuropil regions. Relatively weaker expression in cell bodies. |
| **Rab32** | Strong expression in the visual system, with relative enrichment in the synaptic neuropils lamina and medulla. Relatively weaker and even expression in central neuropils and relatively weaker expression in cell bodies. |
| **RabX1** | Intermediate expression in both cell bodies and most or all synaptic neuropils. |
| **RabX6** | Intermediate expression in both cell bodies and most or all synaptic neuropils. |
| **Rab40** | Intermediate expression in most or all synaptic neuropils. Relatively weaker expression in cell bodies. |
| **Rab23** | Strongly expressed in cells distal and proximal of the lamina and the protocerebral bridge in the central brain. Weak expression in most other neuropils and cell bodies. |
| **Rab21** | Intermediate and relatively even expression in most synaptic neuropils. Weakly expressed in cell bodies. |
| **Rab9** | Intermediate to weak expression in synaptic neuropils and cell bodies. |
| **Rab4** | Strongly expressed in both synaptic neuropils and cell bodies. Particularly enriched in the mushroom body and the medulla. |
