# Supplementary material for: Systematic functional analysis of rab GTPases reveals limits of neuronal robustness to environmental challenges in flies
Source: eLife. 2021 Mar 5;10:e59594. doi: 10.7554/eLife.59594 (PMC8016483; doi:10.7554/eLife.59594)
Supplement: Supplementary file 2. — For the human protein atlas (www.proteinatlas.org based on Fagerberg et al., 2014) 27 tissues were analyzed. The data was summarized in the following way: “ubiquitous” (detected in all tissue/region/cell types), “widespread” (detected in at least a third but not all tissue/region/cell types), “restricted” (detected in more than one but less than one third of tissue/region/cell types). The classifications “tissue specific”, “tissue enriched”, “group enriched” and “uncertain” were used as described in the human protein atlas. Regarding the data of the mouse embryo (E 14.5) transcriptome atlas (www.eurexpress.org based on Diez-Roux et al., 2011) the original classifications were adopted: “regional signal” (signal detected in a limited number of discrete locations), “no regional signal” (in all tissues or not detectable) or “not detected”. Out of the analyzed tissues “brain, spinal cord, CNS nerves, peripheral nervous system, ganglia” were grouped as nervous system and “gut, stomach, liver, pancreas” as intestines. For the flyatlas2 (www.flyatlas.gla.ac.uk, see also based on Leader et al., 2018) only data of female adults were considered. “Head, brain and thoracicoabdominal ganglion” were grouped as “nervous system high”. The following abbreviations were used: human (H), rodent (R), Drosophila melanogaster (Dm), embryo (E), larva (L), adult (A), Mus musculus (Mm), Rattus norvegicus (Rn), Oryctolagus cuniculus (Oc), cell culture (CC). Asterisks indicate if the Rab is specific to Hominidae (*), specific to primates (**) or specific to primates and dolphins (***). [file elife-59594-supp2.docx]

**Supplementary Table / Supplementary File 2**

| **Mammalian Rabs** | **Rab tissue localization in**  **human** | **Rab tissue localization in rodent** | ***D. melanogaster* Rabs** | **Rab tissue localization in *D. melanogaster*** | **References** |
| --- | --- | --- | --- | --- | --- |
| Rab1a | **RNA:** low tissue specificity - ubiquitous  **protein:** general cytoplasmic expression | **RNA:** no regional signal (Mm, E) | DmRab1  (omelette) | **RNA:** ubiquitous (E, A)  **protein:** ubiquitous (L), brain (A), heart (A) | **H**: The human protein atlas  **R:** Transcriptome Atlas mouse embryo  **Dm:** (Chan et al., 2011; Zhang et al., 2007) (Cammarato et al., 2011), flyatlas2 |
| Rab1b | **RNA:** Low tissue specificity – ubiquitous  **protein:** general cytoplasmic expression | **RNA:** no regional signal (Mm, E) |  |  | **H:** The human protein atlas  **R:** Transcriptome Atlas mouse embryo |
| Rab2a | **RNA:** Low tissue specificity – ubiquitous  **protein:** general cytoplasmic expression | **RNA:** no regional signal (Mm, E) | DmRab2 | **RNA:** central nervous system (E), ubiquitous (A)  **protein**: ubiquitous (L), brain (A), heart (A) | **H:** The human protein atlas  **R:** Transcriptome Atlas mouse embryo  **Dm:** (Cammarato et al., 2011; Chan et al., 2011; Zhang et al., 2007), flyatlas2 |
| Rab2b | **RNA:** Low tissue specificity – ubiquitous | **RNA:** weak regional signal in the nervous system (Mm, E) |  |  | **H:** The human protein atlas  **R:** Transcriptome Atlas mouse embryo |
| Rab3a | **RNA:** tissue enriched (brain) -widespread in low levels  **protein:** selective expression in CNS, islets of Langerhans and adrenal medulla | **RNA:** brain (Mm, A and Rn, A) | DmRab3 | **RNA:** central nervous system (E), nervous system high (A)  **protein:** nervous system high (L), brain (A) | **H:** The human protein atlas  **R:** (Elferink et al., 1992; Sollner et al., 2017)  **Dm:** (Chan et al., 2011; Zhang et al., 2007), flyatlas2 |
| Rab3b | **RNA:** tissue enhanced (brain, placenta, prostate) - widespread  **protein:** cytoplasmic expression in pancreatic islet cells, glandular cells in prostate and enteroendocrine cells of the gastrointestinal tract | **RNA:** No regional signal (Mm, E) |  |  | **H:** The human protein atlas  **R:** Transcriptome Atlas mouse embryo |
| Rab3c | **RNA:** group enriched (adrenal gland, brain, pituitary gland) - widespread  **protein:** cytoplasmic expression in CNS, adrenal medulla and islets of Langerhans. | **RNA:** strong regional signal in nervous system and weak in nose (Mm, E) |  |  | **H:** The human protein atlas  **R:** Transcriptome Atlas mouse embryo |
| Rab3d  (Rab16) | **RNA:** Low tissue specificity – ubiquitous  **protein:** cytoplasmic expression in most tissues. | **RNA:** moderate regional signal in nervous system and skin (Mm, E) |  |  | **H:** The human protein atlas  **R:** Transcriptome Atlas mouse embryo |
| Rab4a | **RNA:** Low tissue specificity – ubiquitous | **RNA:** moderate regional signal in the nervous system (Mm, E) | DmRab4 | **RNA:** ubiquitous (E, A), enriched in mesoderm and ectoderm (E)  **protein:** nervous system high (L), brain (A), heart (A) | **H:** The human protein atlas  **R:** Transcriptome Atlas mouse embryo  **Dm:** (Cammarato et al., 2011; Chan et al., 2011; Zhang et al., 2007), flyatlas2 |
| Rab4b | **RNA:** Low tissue specificity – ubiquitous | **RNA:** strong regional signal (nose, alimentary system, skeleton) (Mm, E) |  |  | **H:** The human protein atlas  **R:** Transcriptome Atlas mouse embryo |
| Rab5a | **RNA:** Low tissue specificity – ubiquitous  **protein:** cytoplasmic expression in all tissues | no data | DmRab5 | **RNA:** ubiquitous (E, A), enriched in garland cells (E)  **protein:** ubiquitous (L), brain (A) | **H:** The human protein atlas  **Dm:** (Chan et al., 2011; Zhang et al., 2007), flyatlas2 |
| Rab5b | **RNA:** Low tissue specificity – ubiquitous  **protein:** ubiquitous cytoplasmic expression | no data |  |  | **H:** The human protein atlas |
| Rab5c | **RNA:** Low tissue specificity – ubiquitous  **protein**: cytoplasmic expression in several tissues | **RNA:** not detected (Mm, E) |  |  | **H:** The human protein atlas  **R**: Transcriptome Atlas mouse embryo |
| Rab6a | **RNA:** Low tissue specificity – ubiquitous  **protein**: ubiquitous | **RNA**: strong regional signal in the nervous and alimentary system, eye and nose (Mm, E)  **protein**: ubiquitously expressed (Mm, E) | DmRab6  (warthog) | **RNA:** ubiquitous (E, A)  **protein:** ubiquitous (L), brain (A) | **H**: The human protein atlas  **R**: Transcriptome Atlas mouse embryo, (Bardin et al., 2015)  **Dm**: (Chan et al., 2011; Zhang et al., 2007), flyatlas2 |
| Rab6b | **RNA:** Group enriched (brain, parathyroid gland) – widespread  **protein**: predominantly expressed in the brain | **RNA:** strong regional signal in the nervous system, eye and nose (Mm, E) |  |  | **H**: The human protein atlas, (Opdam et al., 2000)  **R**: Transcriptome Atlas mouse embryo |
| Rab6a’ | **protein**: ubiquitous expression | **protein**: ubiquitously expressed (Mm, E) |  |  | **H**: (Echard et al., 2000)  **R**: (Bardin et al., 2015) |
| Rab6c* | **RNA**: tissue enhanced (parathyroid gland) – widespread;  fetal and adult brain, prostate, testis, and spinal cord | not in mouse |  |  | **H**: The human protein atlas, (Young et al., 2010) |
| Rab6d (Rab41) | See Rab41 | not in mouse |  |  |  |
| Rab7a | **RNA**: Low tissue specificity – ubiquitous  **protein**: Ubiquitous cytoplasmic expression, high expression in skeletal muscle | **RNA**: ubiquitous expression, with high levels in liver, heart and kidney (Mm, E) | DmRab7 | **RNA:** ubiquitous (E, A)  **protein:** ubiquitous (L), brain (A), heart (A) | **H**: The human protein atlas, (Verhoeven et al., 2003)  **R**: Transcriptome Atlas mouse embryo, (Verhoeven et al., 2003)  **Dm**: (Cammarato et al., 2011; Chan et al., 2011; Zhang et al., 2007), flyatlas2 |
| Rab7b | **RNA:** Tissue enhanced (adipose tissue, skin) – widespread | no data |  |  | **H**: The human protein atlas |
| Rab8a | **RNA**: Low tissue specificity – ubiquitous | no data | DmRab8 | **RNA:** ubiquitous (E+A), enriched in mesoderm and ectoderm (E)  **protein:** ubiquitous (L), brain (A) | **H**: The human protein atlas  **Dm**: (Chan et al., 2011; Zhang et al., 2007), flyatlas2 |
| Rab8b | **RNA**: Low tissue specificity – ubiquitous | **RNA**: no regional signal (Mm, E) |  |  | **H**: The human protein atlas  **R**: Transcriptome Atlas mouse embryo |
| Rab8c (Rab13) | See Rab13 | See Rab13 |  |  |  |
| Rab9a | **RNA**: Low tissue specificity – ubiquitous  **protein**: Ubiquitous cytoplasmic expression, most abundant in glandular and lymphoid cells | **RNA**: no regional signal (Mm, E) | DmRab9 | **RNA:** ubiquitous (E+A), enriched in CNS (E)  **protein:** nervous system high (L), brain (A) | **H**: The human protein atlas  **R**: Transcriptome Atlas mouse embryo  **Dm**: (Chan et al., 2011; Zhang et al., 2007), flyatlas2 |
| Rab9b | **RNA**: Tissue enhanced (heart muscle) – widespread  **protein**: Membranous and cytoplasmic expression in most tissues, highest expression in intercalated discs of heart myocytes | **RNA**: no regional signal (Mm, E) |  |  | **H**: The human protein atlas  **R**: Transcriptome Atlas mouse embryo |
| Rab10 | **RNA**: Low tissue specificity – ubiquitous  **protein**: Ubiquitous cytoplasmic expression | RNA: no regional signal (Mm, E) | DmRab10 | **RNA:** ubiquitous (E+A), enriched in CNS (E)  **protein:** widespread (L), brain (A), heart (A) | **H**: The human protein atlas  **R**: Transcriptome Atlas mouse embryo  **Dm**: (Cammarato et al., 2011; Chan et al., 2011; Zhang et al., 2007), flyatlas2, |
| Rab11a | **RNA**: Low tissue specificity -ubiquitous  **protein**: Ubiquitous cytoplasmic and membranous expression | **RNA**: no regional signal (Mm, E) | DmRab11 | **RNA:** ubiquitous (E+A), enriched in gut (E)  **protein:** ubiquitous (L), brain (A), heart (A) | **H**: The human protein atlas  **R**: Transcriptome Atlas mouse embryo  **Dm**:(Cammarato et al., 2011; Chan et al., 2011; Zhang et al., 2007) flyatlas2 |
| Rab11b | **RNA**: Low tissue specificity – ubiquitous | **RNA**: no regional signal (Mm, E) |  |  | **H**: The human protein atlas  **R**: Transcriptome Atlas mouse embryo |
| Rab11c (Rab25) | See Rab25 | See Rab25 |  |  |  |
| Rab12 | **RNA**: Tissue enhanced (skeletal muscle) – ubiquitous  **protein**: Ubiquitous cytoplasmic expression | **RNA**: no regional signal (Mm, E) | - | - | **H**: The human protein atlas  **R**: Transcriptome Atlas mouse embryo |
| Rab13 (Rab8c) | **RNA**: Low tissue specificity – ubiquitous  **protein**: Cytoplasmic expression in most tissues | **RNA**: moderate regional signal brain and spinal cord (Mm, E) | DmRab8 | see DmRab8 | **H**: The human protein atlas  **R**: Transcriptome Atlas mouse embryo |
| Rab14 | **RNA**: Low tissue specificity – ubiquitous  **protein**: General cytoplasmic expression, ubiquitous | **RNA**: no regional signal (Mm, E)  **protein**: ubiquitous expression with highest levels in brain, kidney, spleen and thymus (Rn) | DmRab14 | **RNA:** ubiquitous (E+A), enriched in salivary gland and CNS (E)  **protein:** widespread(L), brain (A) | **H**: The human protein atlas, (Junutula et al., 2004)  **R**: Transcriptome Atlas mouse embryo, (Junutula et al., 2004)  **Dm**: (Chan et al., 2011; Zhang et al., 2007), flyatlas2 |
| Rab15 | **RNA**: tissue enhanced (brain) – widespread  **protein**: Cytoplasmic expression in all tissues (uncertain) | **RNA**:  Specifically expressed in brain (Rn) | - | - | **H**: The human protein atlas  **R:** (Elferink et al., 1992) |
| Rab16 (Rab3d) | See Rab3d | See Rab3d | See DmRab3 |  |  |
| Rab17 | **RNA**: Tissue enhanced (intestine, liver) – widespread  **protein**: Cytoplasmic and membranous expression in several different tissue types, including CNS and most glandular cells | **RNA**: moderate regional signal in nose, alimentary system and salivary gland (Mm, E), specific to epithelial cells, in tissue like kidney, liver and intestine (Mm, A)  **protein**: kidney (basolateral plasma membrane and to apical tubules) (Mm, A) | - | - | **H**: The human protein atlas  **R**: Transcriptome Atlas mouse embryo,(Lutcke et al., 1993) |
| Rab18 | **RNA**: low tissue specificity - ubiquitous  **protein**: Ubiquitous cytoplasmic expression (uncertain) | **RNA**: no regional signal (Mm, E), detected in kidney, liver, intestine, brain, lung, spleen, heart (Mm, A)  **protein**: kidney cortex, tubular structures (Mm) | DmRab18 | **RNA:** ubiquitous (E+A)  **protein:** widespread (L), brain(A), heart (A) | **H**: The human protein atlas  **R**: Transcriptome Atlas mouse embryo, (Lutcke et al., 1994)  **Dm**: (Cammarato et al., 2011; Chan et al., 2011; Zhang et al., 2007), flyatlas2 |
| Rab19a | **RNA**: Tissue enhanced (pancreas)- widespread | **RNA**: not detected in embryo, tissue specifically (Mm, E), intestine lung and spleen, kidney (Mm, A) | DmRab19 | **RNA:** ubiquitous(E+A)  **protein:** nervous system high (L), brain (A) | **H**: The human protein atlas  **R**: Transcriptome Atlas mouse embryo, (Lutcke et al., 1995)  **Dm**: (Chan et al., 2011; Zhang et al., 2007), flyatlas2, this study |
| Rab19b (Rab43) | See Rab43 | See Rab43 |  |  |  |
| Rab20 | **RNA**: Low tissue specificity – ubiquitous  **protein**: Cytoplasmic expression in several tissues (uncertain) | **RNA**: not detected in embryo (Mm, E), in a variety of adult mouse tissue: kidney, liver, lung, spleen, heart but not detected in the brain (Mm, A)  **protein**: kidney (Mm) |  | - | **H**: The human protein atlas  **R**: Transcriptome Atlas mouse embryo,(Lutcke et al., 1994) |
| Rab21 | **RNA**: Low tissue specificity – ubiquitous  **protein**: General cytoplasmic expression | **RNA**: moderate regional signal in nervous, alimentary and haemolymphoid system and eye (Mm, E) | DmRab21 | **RNA:** ubiquitous (E+A), enriched in gut (E)  **protein:** nervous system high (L), brain (A) | **H**: The human protein atlas  **R**: Transcriptome Atlas mouse embryo  **Dm**: (Chan et al., 2011; Zhang et al., 2007), flyatlas2, this study |
| Rab22a | **RNA**: Low tissue specificity – ubiquitous  **protein**: Cytoplasmic expression in most tissues | **RNA**: no regional signal (Mm, E) | - | - | **H**: The human protein atlas  **R**: Transcriptome Atlas mouse embryo |
| Rab22b  (Rab31) | **RNA**: Low tissue specificity – ubiquitous  **protein**: Cytoplasmic expression in most tissues | **RNA**: moderate regional signal in the nervous and alimentary system, salivary gland, skeletal muscles, and skin (Mm, E)  **protein**: enriched in the brain (Mm, A and Rn, A) spleen, and intestine, in much lower levels in other organs (Rn) | - | - | **H**: The human protein atlas  **R**: Transcriptome Atlas mouse embryo, (Chua et al., 2014; Ng et al., 2007) |
| Rab23 | **RNA**: Tissue enhanced (smooth muscle, urinary bladder) – widespread  **protein**: General cytoplasmic and membranous expression | **RNA**: Predominantly brain-enriched (Mm, A)  **protein**: predominantly brain-enriched, low levels in multiple tissues (Mm, A) | DmRab23 | **RNA:** stripes (E)  **protein:** nervous system high (L), brain (A) | **H**: The human protein atlas  **R**: (Guo et al., 2006; Olkkonen et al., 1994)  **Dm**: (Chan et al., 2011; Zhang et al., 2007), this study |
| Rab24 | **RNA**: Low tissue specificity – ubiquitous | **RNA**: regional signal in peripheral nervous system and skin (Mm, E) | - | - | **H**: The human protein atlas  **R**: Transcriptome Atlas mouse embryo |
| Rab25  (Rab11c) | **RNA**: Tissue enhanced (esophagus) – widespread  **protein**: Membranous expression in most epithelial cells | **RNA**: moderate regional signal in nose, alimentary system, salivary gland, intestines, renal/urinary system and skin (Mm, E), specifically expressed in epithelial cells (Oc) | See DmRab11 |  | **H**: The human protein atlas  **R**: Transcriptome Atlas mouse embryo, (Goldenring et al., 1993) |
| Rab26a | **RNA**: Tissue enhanced (brain, liver, pancreas, salivary gland) – widespread | **protein**: parotid gland (Rn, A) | DmRab26 | **RNA:** CNS (E), nervous system high (A)  **protein:** nervous system high (L), brain (A) | **H**: The human protein atlas,(Jin and Mills, 2014)  **R**: (Yoshie et al., 2000)  **Dm**: (Chan et al., 2011; Zhang et al., 2007), flyatlas2, this study |
| Rab26b  (Rab37) | See Rab37 | See Rab37 |  |  |  |
| Rab27a | **RNA**: Low tissue specificity – ubiquitous  **protein**: Cytoplasmic expression in most tissues, including immune cells | **RNA**: strong regional signal in brain, peripheral nervous, haemolymphoid and alimentary system and adrenal gland (Mm, E)  **protein**: broad expression with high levels in large intestine, spleen, eye, lung, stomach, and platelets (Mm) | DmRab27 | **RNA:** ubiquitous (E), nervous system high and ovary (A)  **protein:** nervous system high (L), brain (A) | **H**: The human protein atlas  **R**: Transcriptome Atlas mouse embryo, (Barral et al., 2002)  **Dm**: (Chan et al., 2011; Zhang et al., 2007), flyatlas2, this study |
| Rab27b | **RNA**: Tissue enhanced (brain, stomach) – widespread  **protein**: Cytoplasmic and membranous expression mainly in glandular cells of gastrointestinal tract, breast, salivary gland, prostate, cells in renal tubules and urothelial cells | **RNA**: strong regional signal in the ear, eye, nose, intestines, nervous, alimentary respiratory renal/urinary, reproductive system and limb (Mm, E)  **protein**: platelets, gastrointestinal tract (Mm), pancreas (Rn and Mm, A), bladder, spleen, and brain (Mm, A) |  |  | **H**: The human protein atlas  **R**: Transcriptome Atlas mouse embryo, (Barral et al., 2002; Chen et al., 2004; Chen et al., 2003b; Zhao et al., 2002) |
| Rab28 | **RNA**: Low tissue specificity – ubiquitous  **protein**: Cytoplasmic expression in most tissues | **RNA**: strong regional signal in nose, skeleton, limb (Mm, E) | - | - | **H**: The human protein atlas  **R**: Transcriptome Atlas mouse embryo |
| Rab29 | **RNA**: Low tissue specificity, ubiquitous  **protein**: Cytoplasmic expression in most tissues | no data | - | - | **H**: The human protein atlas |
| Rab30 | **RNA**: Low tissue specificity – ubiquitous  **protein**: Cytoplasmic expression in most tissues | no data | DmRab30 | **RNA:** ubiquitous (E+A), enriched in CNS (E)  **protein:** ubiquitous (L), brain (A) | **H**: The human protein atlas  **R**: Transcriptome Atlas mouse embryo  **Dm**: (Jin et al., 2012; Zhang et al., 2007), flyatlas2, this study |
| Rab31 (Rab22b) | see Rab22b | See Rab22b | - | - |  |
| Rab32a | **RNA:** Tissue enhanced (bone marrow) – ubiquitous  **protein:** Cytoplasmic expression in most tissues | **RNA**:  Broad expression with high levels in the liver (Mm, E) | DmRab32  (lightoid) | **RNA:** malpighian tubules(E), ubiquitous and eye enriched (A)  **protein:** nervous system high (L), brain (A) | **H**: The human protein atlas  **R**: (Cohen-Solal et al., 2003)  **Dm**: (Chan et al., 2011; Zhang et al., 2007), flyatlas2, this study |
| Rab32b (Rab38) | See Rab38 | See Rab38 |  |  |  |
| Rab33a | **RNA:** Tissue enhanced (blood, brain) – widespread | **RNA**:  Expression restricted to brain, weak in ovary and thymus (Mm) | - | - | **H**: The human protein atlas  **R**: (Zheng et al., 1997) |
| Rab33b | **RNA**: Low tissue specificity – ubiquitous | **RNA**:  ubiquitous expression (Mm) | - | - | **H**: The human protein atlas  **R**: (Zheng et al., 1998) |
| Rab34 | **RNA**: Low tissue specificity – ubiquitous  **protein**: General cytoplasmic expression | RNA: no regional signal (Mm, E) | - | - | **H**: The human protein atlas  **R**: Transcriptome Atlas mouse embryo |
| Rab35 | **RNA**: Low tissue specificity – ubiquitous  **protein**: Cytoplasmic expression in most | no data | DmRab35 | **RNA:** ubiquitous (E+A), enriched in CNS (E)  **protein:** ubiquitous (L), brain (A) | **H**: The human protein atlas  **Dm**: (Chan et al., 2011; Zhang et al., 2007), flyatlas2 |
| Rab36 | **RNA**: Tissue enhanced (fallopian tube) – widespread | **RNA**: not detected (Mm, E) | - |  | **H**: The human protein atlas  **R**: Transcriptome Atlas mouse embryo |
| Rab37 | **RNA**: Tissue enhanced (blood, brain), widespread | **RNA**: not detected (Mm, E), bone marrow mast cells (Mm, CC) | DmRab26 | See DmRab26 | **H**: The human protein atlas  **R**: Transcriptome Atlas mouse embryo, (Masuda et al., 2000) |
| Rab38 | **RNA**: Tissue enhanced (retina, tongue)- widespread | **RNA**: strong regional signal in ear, nose, alimentary and respiratory system, salivary gland, stomach and gut, skeleton and skin (Mm, E) | DmRab32 | See DmRab32 | **H**: The human protein atlas  **R**: Transcriptome Atlas mouse embryo |
| Rab39a | **RNA**: Tissue enhanced (brain, pituitary gland and epithelial cells) – widespread | **RNA**: Not detected (Mm, E) | DmRab39 | **RNA:** ubiquitous (E+A)  **protein:** widespread (L), brain (A) | **H**: The human protein atlas, (Chen et al., 2003a) Chen et al., 2003  **R**: Transcriptome Atlas mouse embryo  **Dm**: (Chan et al., 2011; Zhang et al., 2007), flyatlas2 |
| Rab39 b | **RNA**: Tissue enhanced (brain) – widespread  **protein**: Cytoplasmic and membranous expression in several tissues, including cerebral cortex | **RNA**: strong regional signal in the peripheral nervous system, ganglia (Mm, E), brain specific (Mm, A) |  |  | **H**: The human protein atlas,(Giannandrea et al., 2010)  **R**: Transcriptome Atlas mouse embryo, (Giannandrea et al., 2010) |
| Rab40a** | **RNA**: Tissue enhanced (epididymis) – widespread | not in mouse | DmRab40 | **RNA:** ubiquitous (E+A), enriched in CNS (E)  **protein:** nervous system high (L), brain (A) | **H**: The human protein atlas  **Dm**: (Jin et al., 2012; Zhang et al., 2007), flyatlas2, this study |
| Rab40b | **RNA**: Tissue enhanced (brain) - ubiquitous | **RNA**: brain, inner ear and heart tissues (Mm, E+A) |  |  | **H**: The human protein atlas  **R**: (Bedoyan et al., 2012) |
| Rab40al**  (RLGP) | **RNA**: Tissue enhanced (epididymis); human fetal and adult brain and kidney, and adult lung, heart, liver and skeletal muscle | not in mouse |  |  | **H**: The human protein atlas, (Bedoyan et al., 2012) |
| Rab40c | **RNA**: Tissue enhanced (pancreas) – ubiquitous | **RNA**: no regional signal |  |  | **H**: The human protein atlas  **R**: Transcriptome Atlas mouse embryo |
| Rab41 (Rab6d) *** | **RNA**: Tissue enhanced (brain, testis) - restricted | not in mouse | DmRab6 | See DmRab6 | **H**: The human protein atlas |
| Rab42 (Rab39c) | **RNA**: Tissue enhanced (lymphoid tissue) - widespread | no data | DmRab39 | See DmRab39 | **H**: The human protein atlas |
| Rab43 (Rab19b) | **RNA**: Tissue enriched (liver) – ubiquitous  **protein**: Cytoplasmic expression in several tissues. Strong positivity in colloid in thyroid gland as well | no data | DmRab19 | See DmRab19 | **H**: The human protein atlas |
| Rab44 | **RNA**: Tissue enhanced (blood, bone marrow) – widespread | **RNA**: highly expressed in the bone marrow and slightly expressed in the epididymis,  lung, skin, spleen, thymus, ovary, uterus, and liver (Mm, CC)  **protein**: bone marrow, spleen and thymus (Mm, CC) | -- | - | **H**: The human protein atlas  **R**: (Tokuhisa et al., 2020) |
| Rab45 | **RNA**: Low tissue specificity – widespread  **protein**: General cytoplasmic expression | no data | - | - | **H**: The human protein atlas |
| - | - | - | DmRabX1  (chrowded) | **RNA:** ubiquitous (A)  **protein:** nervous system high (L), brain (A) | **Dm**: (Chan et al., 2011), flyatlas2, this study |
| - | - | - | DmRabX4 | **RNA:** CNS(E), nervous system high (A)  **protein:** nervous system high (L), brain (A) | **Dm**: (Chan et al., 2011; Zhang et al., 2007), flyatlas2, this study |
| - | - | - | DmRabX6 | **RNA:** ubiquitous (E+A)  **protein:** nervous system high (L), brain (A) | **Dm**: (Jin et al., 2012; Zhang et al., 2007), flyatlas2, this study |

**References – Supplementary Table 2**

Bardin, S., Miserey-Lenkei, S., Hurbain, I., Garcia-Castillo, D., Raposo, G., and Goud, B. (2015). Phenotypic characterisation of RAB6A knockout mouse embryonic fibroblasts. Biol Cell *107*, 427-439.

Barral, D.C., Ramalho, J.S., Anders, R., Hume, A.N., Knapton, H.J., Tolmachova, T., Collinson, L.M., Goulding, D., Authi, K.S., and Seabra, M.C. (2002). Functional redundancy of Rab27 proteins and the pathogenesis of Griscelli syndrome. J Clin Invest *110*, 247-257.

Bedoyan, J.K., Schaibley, V.M., Peng, W., Bai, Y., Mondal, K., Shetty, A.C., Durham, M., Micucci, J.A., Dhiraaj, A., Skidmore, J.M.*, et al.* (2012). Disruption of RAB40AL function leads to Martin--Probst syndrome, a rare X-linked multisystem neurodevelopmental human disorder. J Med Genet *49*, 332-340.

Cammarato, A., Ahrens, C.H., Alayari, N.N., Qeli, E., Rucker, J., Reedy, M.C., Zmasek, C.M., Gucek, M., Cole, R.N., Van Eyk, J.E.*, et al.* (2011). A mighty small heart: the cardiac proteome of adult Drosophila melanogaster. PLoS One *6*, e18497.

Chan, C.C., Scoggin, S., Wang, D., Cherry, S., Dembo, T., Greenberg, B., Jin, E.J., Kuey, C., Lopez, A., Mehta, S.Q.*, et al.* (2011). Systematic discovery of Rab GTPases with synaptic functions in Drosophila. Curr Biol *21*, 1704-1715.

Chen, T., Han, Y., Yang, M., Zhang, W., Li, N., Wan, T., Guo, J., and Cao, X. (2003a). Rab39, a novel Golgi-associated Rab GTPase from human dendritic cells involved in cellular endocytosis. Biochem Biophys Res Commun *303*, 1114-1120.

Chen, X., Li, C., Izumi, T., Ernst, S.A., Andrews, P.C., and Williams, J.A. (2004). Rab27b localizes to zymogen granules and regulates pancreatic acinar exocytosis. Biochem Biophys Res Commun *323*, 1157-1162.

Chen, Y., Guo, X., Deng, F.M., Liang, F.X., Sun, W., Ren, M., Izumi, T., Sabatini, D.D., Sun, T.T., and Kreibich, G. (2003b). Rab27b is associated with fusiform vesicles and may be involved in targeting uroplakins to urothelial apical membranes. Proc Natl Acad Sci U S A *100*, 14012-14017.

Chua, C.E., Goh, E.L., and Tang, B.L. (2014). Rab31 is expressed in neural progenitor cells and plays a role in their differentiation. FEBS Lett *588*, 3186-3194.

Cohen-Solal, K.A., Sood, R., Marin, Y., Crespo-Carbone, S.M., Sinsimer, D., Martino, J.J., Robbins, C., Makalowska, I., Trent, J., and Chen, S. (2003). Identification and characterization of mouse Rab32 by mRNA and protein expression analysis. Biochim Biophys Acta *1651*, 68-75.

Echard, A., Opdam, F.J., de Leeuw, H.J., Jollivet, F., Savelkoul, P., Hendriks, W., Voorberg, J., Goud, B., and Fransen, J.A. (2000). Alternative splicing of the human Rab6A gene generates two close but functionally different isoforms. Mol Biol Cell *11*, 3819-3833.

Elferink, L.A., Anzai, K., and Scheller, R.H. (1992). rab15, a novel low molecular weight GTP-binding protein specifically expressed in rat brain. J Biol Chem *267*, 22693.

Giannandrea, M., Bianchi, V., Mignogna, M.L., Sirri, A., Carrabino, S., D'Elia, E., Vecellio, M., Russo, S., Cogliati, F., Larizza, L.*, et al.* (2010). Mutations in the small GTPase gene RAB39B are responsible for X-linked mental retardation associated with autism, epilepsy, and macrocephaly. Am J Hum Genet *86*, 185-195.

Goldenring, J.R., Shen, K.R., Vaughan, H.D., and Modlin, I.M. (1993). Identification of a small GTP-binding protein, Rab25, expressed in the gastrointestinal mucosa, kidney, and lung. J Biol Chem *268*, 18419-18422.

Guo, A., Wang, T., Ng, E.L., Aulia, S., Chong, K.H., Teng, F.Y., Wang, Y., and Tang, B.L. (2006). Open brain gene product Rab23: expression pattern in the adult mouse brain and functional characterization. J Neurosci Res *83*, 1118-1127.

Jin, E.J., Chan, C.C., Agi, E., Cherry, S., Hanacik, E., Buszczak, M., and Hiesinger, P.R. (2012). Similarities of Drosophila rab GTPases based on expression profiling: completion and analysis of the rab-Gal4 kit. PLoS One *7*, e40912.

Jin, R.U., and Mills, J.C. (2014). RAB26 coordinates lysosome traffic and mitochondrial localization. J Cell Sci *127*, 1018-1032.

Junutula, J.R., De Maziere, A.M., Peden, A.A., Ervin, K.E., Advani, R.J., van Dijk, S.M., Klumperman, J., and Scheller, R.H. (2004). Rab14 is involved in membrane trafficking between the Golgi complex and endosomes. Mol Biol Cell *15*, 2218-2229.

Lutcke, A., Jansson, S., Parton, R.G., Chavrier, P., Valencia, A., Huber, L.A., Lehtonen, E., and Zerial, M. (1993). Rab17, a novel small GTPase, is specific for epithelial cells and is induced during cell polarization. J Cell Biol *121*, 553-564.

Lutcke, A., Olkkonen, V.M., Dupree, P., Lutcke, H., Simons, K., and Zerial, M. (1995). Isolation of a murine cDNA clone encoding Rab19, a novel tissue-specific small GTPase. Gene *155*, 257-260.

Lutcke, A., Parton, R.G., Murphy, C., Olkkonen, V.M., Dupree, P., Valencia, A., Simons, K., and Zerial, M. (1994). Cloning and subcellular localization of novel rab proteins reveals polarized and cell type-specific expression. J Cell Sci *107 ( Pt 12)*, 3437-3448.

Masuda, E.S., Luo, Y., Young, C., Shen, M., Rossi, A.B., Huang, B.C., Yu, S., Bennett, M.K., Payan, D.G., and Scheller, R.H. (2000). Rab37 is a novel mast cell specific GTPase localized to secretory granules. FEBS Lett *470*, 61-64.

Ng, E.L., Wang, Y., and Tang, B.L. (2007). Rab22B's role in trans-Golgi network membrane dynamics. Biochem Biophys Res Commun *361*, 751-757.

Olkkonen, V.M., Peterson, J.R., Dupree, P., Lutcke, A., Zerial, M., and Simons, K. (1994). Isolation of a mouse cDNA encoding Rab23, a small novel GTPase expressed predominantly in the brain. Gene *138*, 207-211.

Opdam, F.J., Echard, A., Croes, H.J., van den Hurk, J.A., van de Vorstenbosch, R.A., Ginsel, L.A., Goud, B., and Fransen, J.A. (2000). The small GTPase Rab6B, a novel Rab6 subfamily member, is cell-type specifically expressed and localised to the Golgi apparatus. J Cell Sci *113 ( Pt 15)*, 2725-2735.

Sollner, J.F., Leparc, G., Hildebrandt, T., Klein, H., Thomas, L., Stupka, E., and Simon, E. (2017). An RNA-Seq atlas of gene expression in mouse and rat normal tissues. Sci Data *4*, 170185.

Tokuhisa, M., Kadowaki, T., Ogawa, K., Yamaguchi, Y., Kido, M.A., Gao, W., Umeda, M., and Tsukuba, T. (2020). Expression and localisation of Rab44 in immune-related cells change during cell differentiation and stimulation. Sci Rep *10*, 10728.

Verhoeven, K., De Jonghe, P., Coen, K., Verpoorten, N., Auer-Grumbach, M., Kwon, J.M., FitzPatrick, D., Schmedding, E., De Vriendt, E., Jacobs, A.*, et al.* (2003). Mutations in the small GTP-ase late endosomal protein RAB7 cause Charcot-Marie-Tooth type 2B neuropathy. Am J Hum Genet *72*, 722-727.

Yoshie, S., Imai, A., Nashida, T., and Shimomura, H. (2000). Expression, characterization, and localization of Rab26, a low molecular weight GTP-binding protein, in the rat parotid gland. Histochem Cell Biol *113*, 259-263.

Young, J., Menetrey, J., and Goud, B. (2010). RAB6C is a retrogene that encodes a centrosomal protein involved in cell cycle progression. J Mol Biol *397*, 69-88.

Zhang, J., Schulze, K.L., Hiesinger, P.R., Suyama, K., Wang, S., Fish, M., Acar, M., Hoskins, R.A., Bellen, H.J., and Scott, M.P. (2007). Thirty-one flavors of Drosophila rab proteins. Genetics *176*, 1307-1322.

Zhao, S., Torii, S., Yokota-Hashimoto, H., Takeuchi, T., and Izumi, T. (2002). Involvement of Rab27b in the regulated secretion of pituitary hormones. Endocrinology *143*, 1817-1824.

Zheng, J.Y., Koda, T., Arimura, Y., Kishi, M., and Kakinuma, M. (1997). Structure and expression of the mouse S10 gene. Biochim Biophys Acta *1351*, 47-50.

Zheng, J.Y., Koda, T., Fujiwara, T., Kishi, M., Ikehara, Y., and Kakinuma, M. (1998). A novel Rab GTPase, Rab33B, is ubiquitously expressed and localized to the medial Golgi cisternae. J Cell Sci *111 ( Pt 8)*, 1061-1069.
