# Supplementary material for: Systematic functional analysis of rab GTPases reveals limits of neuronal robustness to environmental challenges in flies
Source: eLife. 2021 Mar 5;10:e59594. doi: 10.7554/eLife.59594 (PMC8016483; doi:10.7554/eLife.59594)
Supplement: Supplementary file 3. — Mouse knockout models were listed for the mammalian rab GTPase mutants. Among primary publications, the International Mouse Phenotype Consortium (https://www.mousephenotype.org/) was used for information on the viability of mouse knockout models Information on Drosophila mutant viability is based on this study, if not stated otherwise in the table. Only viability / lethality for homozygous mutants was listed. The following abbreviations were used: Drosophila melanogaster (Dm), endoplasmic reticulum (ER), glucose transporter type 4 (GLUT4), insulin-producing cells (IPCs), Jun-N-terminal kinase (JNK), knockout (KO), mammals (M), matrix metalloproteinases (MMP), multivesicular bodies(MVBs), neuromuscular junction (NMJ), planar cell polarity (PCP), plasma membrane (PM), Saccharomyces cerevisiae(Sc), trans-Golgi network (TGN), 37tyrosinase-related protein-1 (Tyrp-1), ventral nerve cord (VNC). Asterisks indicate if the Rab isspecific to Hominidae (*), specific to primates (**) or specific to primates and dolphins (***). [file elife-59594-supp3.docx]

**Supplementary Table / Supplementary File 3**

| **Mammalian Rabs** | ***S. cerevisiae* Rabs** | ***D. melanogaster* Rabs** | **Function** | **Subcellular localization** | **Mutant viability** | **References** |
| --- | --- | --- | --- | --- | --- | --- |
| Rab1a | Ypt1 | DmRab1 (omelette) | **M**: anterograde melanosome transport  **Sc**: ER-Golgi, intra Golgi and ER-autophagosome transport  **Dm**: controls Golgi structure, cytokinesis, ER-to-Golgi transport, modulates insulin-like peptide secretion in IPCs | **M**: ER, Golgi, mature melanosomes  **Sc**: ER, Golgi  **Dm**: Golgi, ER exit sites | **M**: lethal  **Sc**: lethal  **Dm**: lethal | **Function**  **M**: (Ishida et al., 2012)  **Sc**: (Jedd et al., 1995; Lipatova et al., 2012; Segev et al., 1988)  **Dm**: (Cao et al., 2014; Ke et al., 2018; Sechi et al., 2017)  **Subcellular localization**  **M**: (Ishida et al., 2012; Plutner et al., 1991)  **Sc**: (Baker et al., 1990; Segev et al., 1988)  **Dm**: (Cao et al., 2014; Ke et al., 2018; Sechi et al., 2017)  **Viability**  **M:** International Mouse Phenotyping Consortium  **Sc**: (Giaever et al., 2002)  **Dm**: (Thibault et al., 2004) |
| Rab1b |  |  | **M**: vesicular transport ER-Golgi | **M**: Er, Golgi | **M**: no KO model | **Function and subcellular localization**  **M**: (Plutner et al., 1991) |
| Rab2a |  | DmRab2 | **M**: ER-to-Golgi, maturation of pre-Golgi intermediates  **Dm**: ER-to-Golgi transport, endosome-lysosome fusion, endolysosome to autophagosome fusion | **M**: Pre-Golgi intermediates  **Dm**: Golgi, late endosomes, lysosomes | **M**: lethal  **Dm**: lethal | **Function**  **M**: (Tisdale and Balch, 1996; Tisdale et al., 1992)  **Dm**: (Ke et al., 2018; Lorincz et al., 2017; Lund et al., 2018)  **Subcellular localization**  **M**: (Tisdale and Balch, 1996; Tisdale et al., 1992)  **Dm**: (Gillingham et al., 2014; Lund et al., 2018)  **Viability**  **M**: International Mouse Phenotyping Consortium |
| Rab2b |  |  | **M**: ER-to-Golgi, maturation of pre-Golgi intermediates | **M**: Pre-Golgi intermediates | **M**: no KO model  **Dm**: lethal | **Function and subcellular localization**  **M**: (Tisdale and Balch, 1996; Tisdale et al., 1992) |
| Rab3a | - | DmRab3 | **M**: transport of synaptic vesicles to PM (regulated exocytosis)  **Dm**: regulation and maintenance of presynaptic active zone structure | **M**: synaptic and secretory vesicles, chromaffin granules  **Dm**: presynaptic active zone, synapse | **M**: viable  **Dm**: viable | **Function**  **M**: (Fischer von Mollard et al., 1991)  **Dm**: (Ehmann et al., 2014; Graf et al., 2009)  **Subcellular localization**  **M**: (Darchen et al., 1990; Fischer von Mollard et al., 1990; Schluter et al., 2002)  **Dm**: (Chan et al., 2011; Graf et al., 2009)  **Viability**  **M**: (Geppert et al., 1994)  **Dm**: (Graf et al., 2009) |
| Rab3b | - |  | **M**: exocytosis | **M**: synaptic and secretory vesicles | **M**: viable | **Function and subcellular localization**  **M**: (Schluter et al., 2002)  **Viability**  **M**: (Schluter et al., 2004) |
| Rab3c | - |  | **M**: exocytosis | **M**: synaptic and secretory vesicles | **M**: viable | **Function and subcellular localization**  **M**: (Schluter et al., 2002)  **Viability**  **M**: (Schluter et al., 2004) |
| Rab3d (Rab16) | - |  | **M**: exocytosis | **M**: secretory granules (in mast cells) | **M**: viable | **Function and subcellular localization**  **M**: (Tuvim et al., 1999)  **Viability**  **M**: (Riedel et al., 2002) |
| Rab4a |  | DmRab4 | **M**: controls early sorting event in endocytosis, protein recycling to PM  **Dm**: regulation of synapse organization, anterograde vesicle trafficking, trafficking of cell adhesion molecules to cell surface | **M**: early sorting endosomes, recycling endosomes  **Dm**: fast recycling endosomes, sorting endosomes, axon termini | **M**: viable  **Dm**: viable | **Function**  **M**: (Seachrist et al., 2000; van der Sluijs et al., 1992)  **Dm**: (de Madrid et al., 2015; Dey et al., 2017)  **Subcellular localization**  **M**: (Trischler et al., 1999; van der Sluijs et al., 1992)  **Dm**: (de Madrid et al., 2015; Dey et al., 2017; West et al., 2015)  **Viability**  **M**: International Mouse Phenotyping Consortium |
| Rab4b | - |  | **M**: controls early sorting event in endocytosis, protein recycling to PM | **M**: early sorting endosomes, recycling endosomes | **M**: viable | **Function**  **M**: (Seachrist et al., 2000; van der Sluijs et al., 1992)  **Subcellular localization**  **M**: (Trischler et al., 1999; van der Sluijs et al., 1992)  **Viability**  **M**: International Mouse Phenotyping Consortium |
| Rab5a | Ypt51, 52, 53 | DmRab5 | **M**: (lateral) early endosomes fusion (endocytosis)  **Sc**: endocytosis and vacuolar protein sorting,  Ypt51 and Ypt52: MVB biogenesis and sorting  **Dm**: synaptic vesicle recycling, formation of early endosomes, regulation of endocytosis | **M**: PM, early endosomes  **Sc**: early endosomes  **Dm**: early endosomes | **M**: viable  **Sc**: viable  **Dm**: lethal | **Function**  **M**: (Bucci et al., 1992; Gorvel et al., 1991)  **Sc**: (Nickerson et al., 2012; Singer-Kruger et al., 1994)  **Dm**: (Compagnon et al., 2009; Wucherpfennig et al., 2003)  **Subcellular localization**  **M**: (Bucci et al., 1992)  **Sc**: (Singer-Kruger et al., 1994)  **Dm**: (Wucherpfennig et al., 2003)  **Viability**  **M**: (Dickinson et al., 2016)  **Sc**: (Singer-Kruger et al., 1994)  **Dm**: (Wucherpfennig et al., 2003) |
| Rab5b |  |  | **M**: (lateral) early endosomes fusion (endocytosis) | **M**: PM, early endosomes | **M**: viable | **Function**  **M**: (Bucci et al., 1992; Gorvel et al., 1991)  **Subcellular localization**  **M**: (Bucci et al., 1992)  **Viability**  **M**: International Mouse Phenotyping Consortium |
| Rab5c |  |  | **M**: (lateral) early endosomes fusion (endocytosis) | **M**: PM, early endosomes | **M**: lethal | **Function**  **M**: (Bucci et al., 1992; Gorvel et al., 1991)  **Subcellular localization**  **M**: (Bucci et al., 1992)  **Viability**  **M**: International Mouse Phenotyping Consortium |
| Rab6a | Ypt6 | DmRab6 (warthog) | **M**: Golgi-to-ER recycling  **Sc**: Endosome-Golgi, intra Golgi and retrograde Golgi-ER transport, delivery of Atg9 to phagophore assembly site  **Dm**: regulates autophagy and insulin-TOR signaling, regulation of autolysosomal homeostasis, insulin signaling, axon guidance in R7 photoreceptors, apical transport pathway in photoreceptors | **M**: trans-Golgi cisternae, TGN  **Sc**: Golgi  **Dm**: Golgi, cytoplasmic vesicles, lysosomes and autophagosomes | **M**: lethal  **Sc**: viable  **Dm**: lethal | **Function**  **M**: (Young et al., 2005)  **Sc**: (Kawamura et al., 2014; Luo and Gallwitz, 2003; Suda et al., 2013; Yang and Rosenwald, 2016)  **Dm**: (Ayala et al., 2018; Iwanami et al., 2016; Tong et al., 2011)  **Subcellular localization**  **M**: (Antony et al., 1992; Goud et al., 1990; Sun et al., 2007)  **Sc**: (Kawamura et al., 2014)  **Dm**: (Ayala et al., 2018; Tong et al., 2011)  **Viability**  **M**: (Bardin et al., 2015)  **Sc**: (Giaever et al., 2002)  **Dm**: (Purcell and Artavanis-Tsakonas, 1999) |
| Rab6a’ |  |  | **M**: recycling/retrograde traffic endosome-to-Golgi, Golgi-to-ER recycling | **M**: trans-Golgi cisternae, TGN | **M**: lethal | **Function**  **M**: (Mallard et al., 2002; Young et al., 2005)  **Subcellular localization**  **M**: (Antony et al., 1992; Goud et al., 1990; Sun et al., 2007)  **Viability**  **M**: (Bardin et al., 2015) |
| Rab6b |  |  | **M**: Golgi-associated membrane trafficking | **M**: Golgi | **M**: viable | **Function and subcellular localization**  **M**: (Opdam et al., 2000)  **Viability**  **M**: (Nyitrai et al., 2020) |
| Rab6c* |  |  | **M**: cell cycle progression | **M**: centrosome | **M**: no KO model | **Function and subcellular localization**  **M**: (Young et al., 2010) |
| Rab6d (Rab41) |  |  | **M**: Golgi ribbon formation, ER-to-Golgi trafficking | **M**: Golgi | **M**: no KO model | **Function**  **M**: (Liu et al., 2013; Liu et al., 2016)  **Subcellular localization**  **M**: (Liu et al., 2013) |
| Rab7a | Ypt7 | DmRab7 | **M**: maturation MVBs, fusion MVBs to lysosomes, late endocytic membrane trafficking  **Sc**: endocytosis, autophagy  **Dm**: targets endocytic cargo to late endosomes and lysosomes | **M**: late endosomes, lysosomes  **Sc**: late endosomes  **Dm**: late endosomes, maturing endosomes | **M**: lethal  **Sc**: viable  **Dm**: lethal | **Function**  **M**: (Guerra and Bucci, 2016; Meresse et al., 1995; Vitelli et al., 1997)  **Sc**: (Kirisako et al., 1999; Schimmoller and Riezman, 1993)  **Dm**: (Entchev et al., 2000)  **Subcellular localization**  **M**: (Meresse et al., 1995; Vitelli et al., 1997)  **Sc**: (Kirisako et al., 1999; Schimmoller and Riezman, 1993)  **Dm**: (Entchev et al., 2000; Yousefian et al., 2013)  **Viability**  **M**: (Kawamura et al., 2012)  **Sc**: (Giaever et al., 2002)  **Dm**: (Cherry et al., 2013) |
| Rab7b |  |  | **M**: maturation MVBs, fusion MVBs to lysosomes, late endocytic membrane trafficking | **M**: late endosomes, lysosomes | **M**: lethal | **Function**  **M**: (Guerra and Bucci, 2016; Meresse et al., 1995; Vitelli et al., 1997)  **Subcellular localization**  **M**: (Meresse et al., 1995; Vitelli et al., 1997)  **Viability**  **M**: (Kawamura et al., 2012) |
| Rab8a | Sec4 | DmRab8 | **M**: primary cilia formation (ciliogenesis), trafficking from TGN and recycling endosome to PM (exocytosis)  **Sc**: fusion of Golgi vesicles with PM, secretion, assembly of exocyst  **Dm**: synaptic growth responses in NMJ, regulates recycling endosome function, regulates furrow ingression, regulates exocytic trafficking | **M**: Golgi, early endosomes, vesicular structures, PM, recycling endosomes, primary clilia  **Sc**: Golgi secretory vesicles, PM  **Dm**: PM, Golgi, recycling endosomes | **M**: die prematurely  **Sc**: lethal  **Dm**: lethal | **Function**  **M**: (Ang et al., 2004; Huber et al., 1993; Yoshimura et al., 2007)  **Sc**: (Goud et al., 1988; Guo et al., 1999; Salminen and Novick, 1987)  **Dm**: (Mavor et al., 2016; West et al., 2015)  **Subcellular localization**  **M**: (Ang et al., 2004; Chen et al., 2001; Huber et al., 1993; Sato et al., 2007; Yoshimura et al., 2007)  **Sc**: (Goud et al., 1988; Salminen and Novick, 1987)  **Dm**: (Mavor et al., 2016; West et al., 2015)  **Viability**  **M**: (Sato et al., 2007)  **Sc**: (Giaever et al., 2002)  **Dm**: (Giagtzoglou et al., 2012) |
| Rab8b |  |  | **M**: traffic from TGN to PM (exocytosis), ciliogenesis and apical transport | **M**: Golgi, vesicular structures, PM, primary cilia | **M**: viable | **Function**  **M**: (Huber et al., 1993; Sato et al., 2014)  **Subcellular localization**  **M**: (Chen et al., 2001; Huber et al., 1993; Sato et al., 2014)  **Viability**  **M**: (Sato et al., 2014) |
| Rab8c (Rab13) |  |  | **M**: assembly epithelial tight junctions, endocytic recycling to PM, trafficking between recycling endosomes and TGN | **M**: tight junctions, recycling endosomes, TGN | **M**: viable | **Function**  **M**: (Kohler et al., 2004; Nokes et al., 2008)  **Subcellular localization**  **M**: (Nokes et al., 2008; Zahraoui et al., 1994)  **Viability**  **M**: International Mouse Phenotyping Consortium |
| Rab9a | - | DmRab9 | **M**: traffic between late endosomes and TGN  **Dm**: retrograde trafficking from endosomes to TGN | **M**: late endosomes  **Dm**: early and late endosomes, TGN | **M**: viable  **Dm**: viable | **Function**  **M**: (Lombardi et al., 1993)  **Dm**: (Dong et al., 2013)  **Subcellular localization**  **M**: (Lombardi et al., 1993)  **Dm**: (Dong et al., 2013)  **Viability**  **M**: International Mouse Phenotyping Consortium |
| Rab9b | - |  | **M**: traffic between late endosomes and TGN | **M**: late endosomes | **M**: no KO model  **Dm**: viable | **Function and subcellular localization**  **M**: (Lombardi et al., 1993) |
| Rab10 | - | DmRab10 | **M**: transport from basolateral sorting endosomes to common endosomes, exocytosis of GLUT4 vesicles  **Dm**: regulation of basement membrane secretion/ organization | **M**: basolateral sorting endosome, GLUT4 vesicles  **Dm**: cytoplasm, basal follicle cell surface, lateral PM | **M**: lethal  **Dm**: viable | **Function**  **M**: (Babbey et al., 2006; Sano et al., 2007)  **Dm**: (Isabella and Horne-Badovinac, 2016; Lerner et al., 2013)  **Subcellular localization**  **M**: (Babbey et al., 2006; Larance et al., 2005; Sano et al., 2007)  **Dm**: (Isabella and Horne-Badovinac, 2016; Lerner et al., 2013)  **Viability**  **M**: (Lv et al., 2015) |
| Rab11a | Ypt31, 32 | DmRab11 | **M**: recycling from endosome to PM, traffic from TGN to PM  **Sc**: exit from trans Golgi, Golgi-PM transport, endosome-Golgi recycling  **Dm**: endocytic recycling | **M**: recycling endosomes, Golgi  **Sc**: transitional and late Golgi, endosomes  **Dm**: recycling endosomes | **M**: lethal  **Sc**: viable, double mutant lethal  **Dm**: lethal | **Function**  **M**: (Ullrich et al., 1996; Urbe et al., 1993)  **Sc**: (Benli et al., 1996; Chen et al., 2005; Jedd et al., 1997)  **Dm**: (Dollar et al., 2002)  **Subcellular localization**  **M**: (Ullrich et al., 1996; Urbe et al., 1993)  **Sc**: (Chen et al., 2005; Jedd et al., 1997)  **Dm**: (Dollar et al., 2002)  **Viability**  **M**: (Yu et al., 2014)  **Sc**: (Benli et al., 1996)  **Dm**: (Bellen et al., 2004) |
| Rab11b |  |  | **M**: recycling from endosome to PM, traffic from TGN to PM | **M**: recycling endosomes, Golgi | **M**: viable | **Function and subcellular localization**  **M**: (Ullrich et al., 1996; Urbe et al., 1993)  **Viability**  **M**: (D'Agostino et al., 2019) |
| Rab11c (Rab25) |  |  | **M**: apical recycling pathway | **M**: recycling endosomes | **M**: viable | **Function and subcellular localization**  **M**: (Casanova et al., 1999)  **Viability**  **M**: (Nam et al., 2010) |
| Rab12 | - | - | **M**: degradation of Transferrin receptor from recycling endosome to lysosome | **M**: recycling endosome | **M**: viable | **Function and subcellular localization**  **M**: (Matsui et al., 2011)  **Viability**  **M**: International Mouse Phenotyping Consortium |
| Rab13 (Rab8c) | Sec4 | DmRab8 | **M**: assembly epithelial tight junctions, endocytic recycling to PM, trafficking between recycling endosomes and TGN  **Sc**: fusion of Golgi vesicles with PM, secretion, assembly of exocyst  **Dm**: synaptic growth responses in NMJ, regulates recycling endosome function, regulates furrow ingression, regulates exocytic trafficking | **M**: tight junctions, recycling endosomes, TGN  **Sc**: Golgi secretory vesicles, PM  **Dm**: PM, Golgi, recycling endosomes | **M**: viable  **Sc**: lethal  **Dm**: lethal | **Function**  **M**: (Kohler et al., 2004; Nokes et al., 2008)  **Sc**: (Goud et al., 1988; Guo et al., 1999; Salminen and Novick, 1987)  **Dm**: (Mavor et al., 2016; West et al., 2015)  **Subcellular localization**  **M**: (Nokes et al., 2008; Zahraoui et al., 1994)  **Sc**: (Goud et al., 1988; Salminen and Novick, 1987)  **Dm**: (Mavor et al., 2016; West et al., 2015)  **Viability**  **M**: International Mouse Phenotyping Consortium  **Sc**: (Giaever et al., 2002)  **Dm**: (Giagtzoglou et al., 2012) |
| Rab14 | - | DmRab14 | **M**: regulation of carrier membranes between ER/Golgi and endosomes  **Dm**: phagosome maturation /acidification, fusion of phagosomes with late endosomes and lysosomes | **M**: ER, TGN, early endosomes, GLUT4 vesicles  **Dm**: phagosomes, early and late endosomes | **M**: no KO model  **Dm**: viable | **Function**  **M**: (Junutula et al., 2004)  **Dm**: (Garg and Wu, 2014)  **Subcellular localization**  **M**: (Junutula et al., 2004; Larance et al., 2005)  **Dm**: (Garg and Wu, 2014) |
| Rab15 | - | - | **M**: traffic from early/sorting endosome to recycling endosome | **M**: early/sorting endosomes, pericentriolar recycling endosomes | **M**: viable | **Function**  **M**: (Zuk and Elferink, 2000)  **Subcellular localization**  **M**: (Zuk and Elferink, 1999, 2000)  **Viability**  **M**: International Mouse Phenotyping Consortium |
| Rab16 (Rab3d) | - | DmRab3 | **M**: exocytosis  **Dm**: regulation and maintenance of presynaptic active zone structure | **M**: secretory granules (in mast cells)  **Dm**: presynaptic active zone, synapse | **M**: viable  **Dm**: viable | **Function**  **M**: (Tuvim et al., 1999)  **Dm**: (Ehmann et al., 2014; Graf et al., 2009)  **Subcellular localization**  **M**: (Tuvim et al., 1999)  **Dm**: (Chan et al., 2011; Graf et al., 2009)  **Viability**  **M**: (Riedel et al., 2002)  **Dm**: (Graf et al., 2009) |
| Rab17 | - | - | **M**: Transcytosis | **M**: recycling endosomes, basolateral PM, apical tubules | **M**: viable | **Function**  **M**: (Zacchi et al., 1998)  **Subcellular localization**  **M**: (Hunziker and Peters, 1998; Lutcke et al., 1993; Zacchi et al., 1998)  **Viability**  **M**: International Mouse Phenotyping Consortium |
| Rab18 | - | DmRab18 | **M**: lipid droplet formation  **Dm**: unknown | **M**: ER, Golgi, lipid droplets  **Dm**: ER, early Golgi, early endosomes | **M**: viable  **Dm**: viable | **Function**  **M**: (Ozeki et al., 2005)  **Subcellular localization**  **M**: (Dejgaard et al., 2008; Ozeki et al., 2005)  **Dm**: (Chan et al., 2011; Gillingham et al., 2014)  **Viability**  **M**: (Carpanini et al., 2014) |
| Rab19a | - | DmRab19 | **M**: unknown  **Dm**: promotes enteroendocrine cell differentiation (in cooperation with Atg16) | **M**: Golgi  **Dm**: Golgi, recycling endosomes | **M**: viable  **Dm**: viable | **Function**  **Dm**: (Nagy et al., 2017)  **Subcellular localization**  **M**: (Sinka et al., 2008)  **Dm**: (Chan et al., 2011; Gillingham et al., 2014; Sinka et al., 2008)  **Viability**  **M**: (Dickinson et al., 2016) |
| Rab19b (Rab43) | - |  | **M**: retrograde trafficking from endosomes to TGN, biogenesis and maintenance of Golgi structure, anterograde trafficking between ER-to-Golgi, through medial Golgi | **M**: ER, medial Golgi, TGN | **M**: viable  **Dm**: viable | **Function**  **M**: (Cox et al., 2016; Dejgaard et al., 2008; Fuchs et al., 2007; Haas et al., 2007)  **Subcellular localization**  **M**: (Cox et al., 2016; Dejgaard et al., 2008; Fuchs et al., 2007)  **Viability**  **M**: (Dickinson et al., 2016; Kretzer et al., 2016) |
| Rab20 | - | - | **M**: early to late macropinosome maturation | **M**: macropinosomes | **M**: viable | **Function**  **M**: (Egami and Araki, 2012a, b)  **Subcellular localization**  **M**: (Egami and Araki, 2012b)  **Viability**  **M**: (Dickinson et al., 2016) |
| Rab21 | - | DmRab21 | **M**: integrin endocytosis to regulate cell adhesion and cytokinesis  **Dm**: fusion of autophagosomes with lysosomes, autophagosome maturation | **M**: early endosomes  **Dm**: early and late endosomes | **M**: lethal  **Dm**: viable | **Function**  **M**: (Pellinen et al., 2006; Pellinen et al., 2008)  **Dm**: (Jean et al., 2015)  **Subcellular localization**  **M**: (Simpson et al., 2004)  **Dm**: (Jean et al., 2015; Jean et al., 2012)  **Viability**  **M**: International Mouse Phenotyping Consortium |
| Rab22a | - | - | **M**: endosomal trafficking to Golgi | **M**: early endosomes, TGN | **M**: viable | **Function and subcellular localization**  **M**: (Kauppi et al., 2002)  **Viability**  **M**: International Mouse Phenotyping Consortium |
| Rab22b (Rab31) | - | - | **M**: transport of mannose 6-phosphate receptors from TGN to endosomes, TGN organization | **M**: early and late endosomes, TGN | **M**: viable | **Function**  **M**: (Rodriguez-Gabin et al., 2009)  **Subcellular localization**  **M**: (Ng et al., 2007; Rodriguez-Gabin et al., 2001)  **Viability**  **M**: International Mouse Phenotyping Consortium |
| Rab23 | - | DmRab23 | **M**: negative regulator of mouse Sonic Hedgehog signaling pathway, left-right patterning in mouse embryo  **Dm**: restriction of actin accumulation in wing cells, localization of core PCP proteins, regulates PCP, regulates hedgehog ligand trafficking in germline stem cell niche | **M**: PM, early sorting endosomes  **Dm**: cytoplasm, PM | **M**: lethal  **Dm**: viable | **Function**  **M**: (Eggenschwiler et al., 2006; Eggenschwiler et al., 2001; Fuller et al., 2014)  **Dm**: (Cicek et al., 2016; Pataki et al., 2010)  **Subcellular localization**  **M**: (Evans et al., 2003)  **Dm**: (Pataki et al., 2010)  **Viability**  **M**: (Dickinson et al., 2016) |
| Rab24 | - | - | **M**: maturation and/or clearance of autophagic compartments | **M**: ER, cis-Golgi, late endosomes | **M**: viable | **Function**  **M**: (Yla-Anttila et al., 2015)  **Subcellular localization**  **M**: (Olkkonen et al., 1993)  **Viability**  **M**: (Dickinson et al., 2016) |
| Rab25 (Rab11c) | - | DmRab11 | **M**: apical recycling pathway  **Sc**: exit from trans Golgi, Golgi-PM transport, endosome-Golgi recycling  **Dm**: endocytic recycling | **M**: recycling endosomes  **Sc**: transitional and late Golgi, endosomes  **Dm**: recycling endosomes | **M**: viable  **Sc**: viable, double mutant lethal  **Dm**: lethal | **Function**  **M**: (Casanova et al., 1999)  **Sc**: (Benli et al., 1996; Chen et al., 2005; Jedd et al., 1997)  **Dm**: (Dollar et al., 2002)  **Subcellular localization**  **M**: (Casanova et al., 1999)  **Sc**: (Chen et al., 2005; Jedd et al., 1997)  **Dm**: (Dollar et al., 2002)  **Viability**  **M**: (Nam et al., 2010)  **Sc**: (Benli et al., 1996)  **Dm**: (Bellen et al., 2004) |
| Rab26a | - | DmRab26 | **M**: lysosomal traffic, secretion/exocytosis  **Dm**: stimulus-dependent membrane receptor turnover | **M**: secretory granules, lysosomes  **Dm**: synapse | **M**: viable  **Dm**: viable | **Function**  **M**: (Jin and Mills, 2014; Yoshie et al., 2000)  **Dm**: This study  **Subcellular localization**  **M**: (Jin and Mills, 2014; Yoshie et al., 2000)  **Dm**: (Chan et al., 2011)  **Viability**  **M**: (Dong et al., 2018) |
| Rab26b (Rab37) | - |  | **M**: mast cell degranulation/exocytosis of mast cell dense core granules, insulin exocytosis | **M**: (insulin-containing) secretory granules | **M**: no KO model  **Dm**: viable | **Function**  **M**: (Higashio et al., 2016; Ljubicic et al., 2013; Masuda et al., 2000)  **Subcellular localization**  **M**: (Ljubicic et al., 2013; Masuda et al., 2000) |
| Rab27a | - | DmRab27 | **M**: transport of melanosomes to PM/mast cell degranulation, exosome secretion  **Dm**: exosomal secretion | **M**: multivesicular endosomes, melanosomes, melanosome-resident proteins  **Dm**: synapses, synaptic vesicles | **M**: viable  **Dm**: viable | **Function**  **M**: (Hume et al., 2001; Ostrowski et al., 2010)  **Dm**: (Corrigan et al., 2014)  **Subcellular localization**  **M**: (Bahadoran et al., 2001; Hume et al., 2001; Ostrowski et al., 2010)  **Dm**: (Chan et al., 2011)  **Viability**  **M**: (Wilson et al., 2000)  **Dm**: (Chan et al., 2011) |
| Rab27b | - |  | **M**: transport of melanosomes, formation/maintenance of dendritic extensions in melanocytes, exosome secretion, platelet dense granule secretion | **M**: melanosomes, Golgi, TGN and multivesicular endosomes | **M**: viable | **Function**  **M**: (Chen et al., 2002; Ostrowski et al., 2010; Tolmachova et al., 2007)  **Subcellular localization**  **M**: (Chen et al., 2002; Ostrowski et al., 2010)  **Viability**  **M**: (Tolmachova et al., 2007) |
| Rab28 | - | - | **M**: phagocytosis of outer cone segment (in murine retinal pigmented epithelium) | **M**: basal body and ciliary rootlet of photoreceptors | **M**: viable | **Function**  **M**: (Ying et al., 2018)  **Subcellular localization**  **M**: (Roosing et al., 2013)  **Viability**  **M**: (Ying et al., 2018) |
| Rab29 | - | - | **M**: maintenance of TGN, integrity retrograde traffic of Mannose-6-Phosphate receptor, lysosomal trafficking (Golgi-to-lysosome), master regulator of LRRK2 | **M**: TGN | **M**: viable | **Function**  **M**: (MacLeod et al., 2013; Purlyte et al., 2018; Wang et al., 2014)  **Subcellular localization**  **M**: (Wang et al., 2014)  **Viability**  **M**: (Kuwahara et al., 2016) |
| Rab30 | - | DmRab30 | **M**: structural integrity of Golgi apparatus  **Dm**: embryonic and adult morphogenesis (JNK-dependent dorsal closure, embryonic head involution, thorax closure) | **M**: Golgi  **Dm**: Golgi, endosomes, trans-Golgi | **M**: no KO model  **Dm**: viable | **Function**  **M**: (Kelly et al., 2012)  **Dm**: (Thomas et al., 2009)  **Subcellular localization**  **M**: (Kelly et al., 2012)  **Dm**: (Gillingham et al., 2014; Sinka et al., 2008; Thomas et al., 2009) |
| Rab31 (Rab22b) | - | - | **M**: transport of mannose 6-phosphate receptors from TGN to endosomes, TGN organization | **M**: early and late endosomes, TGN | **M**: viable | **Function**  **M**: (Rodriguez-Gabin et al., 2009)  **Subcellular localization**  **M**: (Ng et al., 2007; Rodriguez-Gabin et al., 2001)  **Viability**  **M**: International Mouse Phenotyping Consortium |
| Rab32a | - | DmRab32 (lightoid) | **M**: intracellular sorting of Tyrp-1 and tyrosinase, sorting of melanogenic enzymes from TGN to melanosomes, synchronization of mitochondrial fission  **Dm**: eye pigment granule biosynthesis, maintenance of lipid droplet size, regulation of lipid storage, regulation of autophagy | **M**: mature melanosomes, perinuclear (Tyrp-1 containing) vesicles, mitochondria  **Dm**: lysosomes and autophagosomes | **M**: (sub)viable  **Dm**: viable | **Function**  **M**: (Alto et al., 2002; Wasmeier et al., 2006)  **Dm**: (Ma et al., 2004; Wang et al., 2012)  **Subcellular localization**  **M**: (Alto et al., 2002; Cohen-Solal et al., 2003; Wasmeier et al., 2006)  **Dm**: (Wang et al., 2012)  **Viability**  **M**: (Aguilar et al., 2019; Dickinson et al., 2016)  **Dm**: (Ma et al., 2004) |
| Rab32b (Rab38) | - |  | **M**: melanosome maturation, intracellular sorting of Tyrp-1 and tyrosinase, sorting of melanogenic enzymes from TGN to melanosomes | **M**: mature melanosomes, perinuclear (Tyrp-1 containing) vesicles, mitochondria | **M**: viable | **Function and subcellular localization**  **M**: (Wasmeier et al., 2006)  **Viability**  **M**: (Aguilar et al., 2019) |
| Rab33a | - | - | **M**: anterograde axonal transport of post-Golgi synaptophysin-pos. vesicles | **M**: Golgi, synaptophysin-pos. vesicles | **M**: no KO model | **Function and subcellular localization**  **M**: (Nakazawa et al., 2012) |
| Rab33b | - | - | **M**: modulation of autophagosome formation, retrograde transport Golgi-to-ER | **M**: Golgi apparatus (esp. medial Golgi cisternae) | **M**: viable | **Function**  **M**: (Itoh et al., 2008; Valsdottir et al., 2001)  **Subcellular localization**  **M**: (Zheng et al., 1998)  **Viability**  **M**: International Mouse Phenotyping Consortium |
| Rab34 | - | - | **M**: intracellular lysosomal positioning, macropinosome formation, ciliary vesicle formation | **M**: Golgi, macropinosomes, | **M**: lethal | **Function**  **M**: (Sun et al., 2003; Wang and Hong, 2002; Xu et al., 2018)  **Subcellular localization**  **M**: (Speight and Silverman, 2005; Sun et al., 2003; Wang and Hong, 2002)  **Viability**  **M**: (Dickinson et al., 2016; Xu et al., 2018) |
| Rab35 | - | DmRab35 | **M**: formation of phagosomes (phagocytosis), remodeling of actin cytoskeleton, cadherin-dependent adherens junction formation, controls fast endocytotic recycling pathway, cytokinesis  **Dm**: actin filament assembly during bristle development, vesicle transport during phagocytosis, trafficking from PM to early endosome, cytoskeletal remodeling at PM, endosomal trafficking to synaptic vesicles | **M**: PM, endocytic compartments, near to actin filaments  **Dm**: PM, NMJ boutons, synapses of VNC | **M**: lethal  **Dm**: semi-lethal | **Function**  **M**: (Charrasse et al., 2013; Chevallier et al., 2009; Egami et al., 2011; Kouranti et al., 2006)  **Dm**: (Jewett et al., 2017; Shim et al., 2010; Uytterhoeven et al., 2011; Zhang et al., 2009)  **Subcellular localization**  **M**: (Chevallier et al., 2009; Kouranti et al., 2006)  **Dm**: (Chan et al., 2011; Jewett et al., 2017; Shim et al., 2010; Uytterhoeven et al., 2011)  **Viability**  **M**: (Dickinson et al., 2016) |
| Rab36 | - | - | **M**: regulates spatial distribution of late endosomes and lysosomes, retrograde transport of melanosomes | **M**: Golgi | **M**: viable | **Function**  **M**: (Chen et al., 2010; Matsui et al., 2012)  **Subcellular localization**  **M**: (Chen et al., 2010)  **Viability**  **M**: (Dickinson et al., 2016) |
| Rab37 (Rab26b) | - | DmRab26 | **M**: mast cell degranulation/exocytosis of mast cell dense core granules, insulin exocytosis  **Dm**: stimulus-dependent membrane receptor turnover | **M**: (insulin-containing) secretory granules  **Dm**: synapse | **M**: no KO model  **Dm**: viable | **Function**  **M**: (Higashio et al., 2016; Ljubicic et al., 2013; Masuda et al., 2000)  **Dm**: This study  **Subcellular localization**  **M**: (Ljubicic et al., 2013; Masuda et al., 2000)  **Dm**: (Chan et al., 2011) |
| Rab38 (Rab32b) | - | DmRab32 | **M**: melanosome maturation, intracellular sorting of Tyrp-1 and tyrosinase, sorting of melanogenic enzymes from TGN to melanosomes  **Dm**: eye pigment granule biosynthesis, maintenance of lipid droplet size, regulation of lipid storage, regulation of autophagy | **M**: mature melanosomes, perinuclear (Tyrp-1 containing) vesicles, mitochondria  **Dm**: lysosomes and autophagosomes | **M**: viable  **Dm**: viable | **Function**  **M**: (Wasmeier et al., 2006)  **Dm**: (Ma et al., 2004; Wang et al., 2012)  **Subcellular localization**  **M**: (Wasmeier et al., 2006)  **Dm**: (Wang et al., 2012)  **Viability**  **M**: (Aguilar et al., 2019)  **Dm**: (Ma et al., 2004) |
| Rab39a | - | DmRab39 | **M**: secretion of pro-inflammatory cytokine, phagosome acidification, autophagosome formation  **Dm**: unknown | **M**: late endosomes, lysosomes  **Dm**: Golgi, late endosomes, synapse | **M**: viable  **Dm**: viable | **Function**  **M**: (Becker et al., 2009; Seto et al., 2013; Seto et al., 2011)  **Subcellular localization**  **M**: (Seto et al., 2013)  **Dm**: (Chan et al., 2011; Gillingham et al., 2014; Jin et al., 2012)  **Viability**  **M**: (Cruz et al., 2020) |
| Rab39b | - |  | **M**: function in synaptic activity/transmission, regulates traffic of glutamate receptor subunits to synaptic terminals | **M**: Golgi | **M**: viable | **Function**  **M**: (Mignogna et al., 2015)  **Subcellular localization**:  **M**: (Giannandrea et al., 2010)  **Viability**  **M**: (Gao et al., 2020) |
| Rab39c (Rab42) | - |  | **M**: unknown | **M**: unknown | **M**: no KO model |  |
| Rab40a** | - | DmRab40 | **M**: unknown  **Dm**: unknown | **M**: unknown  **Dm**: synapse and neuronal cell body | **M**: no KO model  **Dm**: viable | **Subcellular localization**  **Dm**: (Jin et al., 2012) |
| Rab40b | - |  | **M**: transport of MMP2/9 secretory vesicles during invadopodia formation, regulation of MMP secretion | **M**: TGN-derived secretory vesicles | **M**: viable | **Function and subcellular localization**  **M**: (Jacob et al., 2013)  **Viability**  **M**: International Mouse Phenotyping Consortium |
| Rab40c | - |  | **M**: modulates biogenesis of lipid droplets, receptor recycling in oligodendrocytes | **M**: lipid droplets, recycling endosomes | **M**: subviable | **Function and subcellular localization**  **M**: (Rodriguez-Gabin et al., 2004; Tan et al., 2013)  **Viability**  **M**: (Dickinson et al., 2016) |
| Rab40aL (RLGP)** | - |  | **M**: unknown | **M**: mitochondria | **M**: no KO model | **Subcellular localization**  **M**: (Bedoyan et al., 2012; Saito-Ohara et al., 2002) |
| Rab41 (Rab6d)*** | Ypt6 | DmRab6 | **M**: Golgi ribbon formation, ER-to-Golgi trafficking  **Sc**: Endosome-Golgi, intra Golgi and retrograde Golgi-ER transport, delivery of Atg9 to phagophore assembly site  **Dm**: regulates autophagy and insulin-TOR signaling, regulation of autolysosomal homeostasis, insulin signaling, axon guidance in R7 photoreceptors, apical transport pathway in photoreceptors | **M**: Golgi  **Sc:** Golgi  **Dm**: Golgi, cytoplasmic vesicles, lysosomes and autophagosomes | **M**: no KO model  **Sc**: viable  **Dm**: lethal | **Function**  **M**: (Liu et al., 2013; Liu et al., 2016)  **Sc**: (Kawamura et al., 2014; Luo and Gallwitz, 2003; Suda et al., 2013; Yang and Rosenwald, 2016)  **Dm**: (Ayala et al., 2018; Iwanami et al., 2016; Tong et al., 2011)  **Subcellular localization**  **M**: (Liu et al., 2013)  **Sc**: (Kawamura et al., 2014)  **Dm**: (Ayala et al., 2018; Tong et al., 2011)  **Viability**  **Sc**: (Giaever et al., 2002)  **Dm**: (Purcell and Artavanis-Tsakonas, 1999) |
| Rab42 (Rab39c) | - | DmRab39 | **M**: unknown  **Dm**: unknown | **M**: unknown  **Dm**: Golgi, late endosomes, synapse | **M**: no KO model  **Dm**: viable | **Subcellular localization**  **Dm**: (Chan et al., 2011; Gillingham et al., 2014; Jin et al., 2012) |
| Rab43 (Rab19b) | - | DmRab19 | **M**: retrograde trafficking from endosomes to TGN, biogenesis and maintenance of Golgi structure, anterograde trafficking between ER-to-Golgi, through medial Golgi  **Dm**: promotes enteroendocrine cell differentiation (in cooperation with Atg16) | **M**: ER, medial Golgi, TGN  **Dm**: Golgi, recycling endosomes | **M**: viable  **Dm**: viable | **Function**  **M**: (Cox et al., 2016; Dejgaard et al., 2008; Fuchs et al., 2007; Haas et al., 2007)  **Dm**: (Nagy et al., 2017)  **Subcellular localization**  **M**: (Cox et al., 2016; Dejgaard et al., 2008; Fuchs et al., 2007)  **Dm**: (Chan et al., 2011; Gillingham et al., 2014; Sinka et al., 2008)  **Viability**  **M**: (Dickinson et al., 2016; Kretzer et al., 2016) |
| Rab44a | - | - | **M**: regulation of osteoclast differentiation, granule exocytosis in mast cells | **M**: Golgi, lysosomes | **M**: viable | **Function**  **M**: (Kadowaki et al., 2020; Yamaguchi et al., 2018)  **Subcellular localization**  **M**: (Tokuhisa et al., 2020; Yamaguchi et al., 2018)  **Viability**  **M**: (Kadowaki et al., 2020) |
| Rab44b | - | - | **M**: regulation of osteoclast differentiation, granule exocytosis in mast cells | **M**: Golgi, lysosomes | **M**: viable | **Function**  **M**: (Kadowaki et al., 2020; Yamaguchi et al., 2018)  **Subcellular localization**  **M**: (Tokuhisa et al., 2020; Yamaguchi et al., 2018)  **Viability**  **M**: (Kadowaki et al., 2020) |
| Rab45 | - | - | **M**: induction of apoptosis in CML progenitor cells | **M**: perinuclear region | **M**: no KO model | **Function**  **M**: (Nakamura et al., 2011)  **Subcellular localization**  **M**: (Shintani et al., 2007) |
| - | - | RabX1 (chrowded) | **Dm**: trafficking between early and late endosomes, formation of endolysosomes | **Dm**: recycling endosomes, late endosomes | **Dm**: viable | **Function**  **Dm**: (Laiouar et al., 2020; Woichansky et al., 2016)  **Subcellular localization**  **Dm**: (Chan et al., 2011; Laiouar et al., 2020) |
| - | - | RabX4 | **Dm**: unknown | **Dm**: synapses, recycling endosomes | **Dm**: viable | **Subcellular localization**  **Dm**: (Chan et al., 2011) |
| - | - | RabX6 | **Dm**: unknown | **Dm**: neuronal cell body | **Dm**: viable | **Subcellular localization**  **Dm**: (Jin et al., 2012) |

* = Rab6c is specific to Hominidae; ** = Rab40a and Rab40aL are specific to primates; *** = Rab41 (Rab6d) is specific to primates and dolphins (Klopper et al., 2012)

**References – Supplementary Table 3**

Aguilar, A., Weber, J., Boscher, J., Freund, M., Ziessel, C., Eckly, A., Magnenat, S., Bourdon, C., Hechler, B., Mangin, P.H.*, et al.* (2019). Combined deficiency of RAB32 and RAB38 in the mouse mimics Hermansky-Pudlak syndrome and critically impairs thrombosis. Blood Adv *3*, 2368-2380.

Alto, N.M., Soderling, J., and Scott, J.D. (2002). Rab32 is an A-kinase anchoring protein and participates in mitochondrial dynamics. J Cell Biol *158*, 659-668.

Ang, A.L., Taguchi, T., Francis, S., Folsch, H., Murrells, L.J., Pypaert, M., Warren, G., and Mellman, I. (2004). Recycling endosomes can serve as intermediates during transport from the Golgi to the plasma membrane of MDCK cells. J Cell Biol *167*, 531-543.

Antony, C., Cibert, C., Geraud, G., Santa Maria, A., Maro, B., Mayau, V., and Goud, B. (1992). The small GTP-binding protein rab6p is distributed from medial Golgi to the trans-Golgi network as determined by a confocal microscopic approach. J Cell Sci *103 ( Pt 3)*, 785-796.

Ayala, C.I., Kim, J., and Neufeld, T.P. (2018). Rab6 promotes insulin receptor and cathepsin trafficking to regulate autophagy induction and activity in Drosophila. J Cell Sci *131*.

Babbey, C.M., Ahktar, N., Wang, E., Chen, C.C., Grant, B.D., and Dunn, K.W. (2006). Rab10 regulates membrane transport through early endosomes of polarized Madin-Darby canine kidney cells. Mol Biol Cell *17*, 3156-3175.

Bahadoran, P., Aberdam, E., Mantoux, F., Busca, R., Bille, K., Yalman, N., de Saint-Basile, G., Casaroli-Marano, R., Ortonne, J.P., and Ballotti, R. (2001). Rab27a: A key to melanosome transport in human melanocytes. J Cell Biol *152*, 843-850.

Baker, K.P., Schaniel, A., Vestweber, D., and Schatz, G. (1990). A yeast mitochondrial outer membrane protein essential for protein import and cell viability. Nature *348*, 605-609.

Bardin, S., Miserey-Lenkei, S., Hurbain, I., Garcia-Castillo, D., Raposo, G., and Goud, B. (2015). Phenotypic characterisation of RAB6A knockout mouse embryonic fibroblasts. Biol Cell *107*, 427-439.

Becker, C.E., Creagh, E.M., and O'Neill, L.A. (2009). Rab39a binds caspase-1 and is required for caspase-1-dependent interleukin-1beta secretion. J Biol Chem *284*, 34531-34537.

Bedoyan, J.K., Schaibley, V.M., Peng, W., Bai, Y., Mondal, K., Shetty, A.C., Durham, M., Micucci, J.A., Dhiraaj, A., Skidmore, J.M.*, et al.* (2012). Disruption of RAB40AL function leads to Martin--Probst syndrome, a rare X-linked multisystem neurodevelopmental human disorder. J Med Genet *49*, 332-340.

Bellen, H.J., Levis, R.W., Liao, G., He, Y., Carlson, J.W., Tsang, G., Evans-Holm, M., Hiesinger, P.R., Schulze, K.L., Rubin, G.M.*, et al.* (2004). The BDGP gene disruption project: single transposon insertions associated with 40% of Drosophila genes. Genetics *167*, 761-781.

Benli, M., Doring, F., Robinson, D.G., Yang, X., and Gallwitz, D. (1996). Two GTPase isoforms, Ypt31p and Ypt32p, are essential for Golgi function in yeast. EMBO J *15*, 6460-6475.

Bucci, C., Parton, R.G., Mather, I.H., Stunnenberg, H., Simons, K., Hoflack, B., and Zerial, M. (1992). The small GTPase rab5 functions as a regulatory factor in the early endocytic pathway. Cell *70*, 715-728.

Cao, J., Ni, J., Ma, W., Shiu, V., Milla, L.A., Park, S., Spletter, M.L., Tang, S., Zhang, J., Wei, X.*, et al.* (2014). Insight into insulin secretion from transcriptome and genetic analysis of insulin-producing cells of Drosophila. Genetics *197*, 175-192.

Carpanini, S.M., McKie, L., Thomson, D., Wright, A.K., Gordon, S.L., Roche, S.L., Handley, M.T., Morrison, H., Brownstein, D., Wishart, T.M.*, et al.* (2014). A novel mouse model of Warburg Micro syndrome reveals roles for RAB18 in eye development and organisation of the neuronal cytoskeleton. Dis Model Mech *7*, 711-722.

Casanova, J.E., Wang, X., Kumar, R., Bhartur, S.G., Navarre, J., Woodrum, J.E., Altschuler, Y., Ray, G.S., and Goldenring, J.R. (1999). Association of Rab25 and Rab11a with the apical recycling system of polarized Madin-Darby canine kidney cells. Mol Biol Cell *10*, 47-61.

Chan, C.C., Scoggin, S., Wang, D., Cherry, S., Dembo, T., Greenberg, B., Jin, E.J., Kuey, C., Lopez, A., Mehta, S.Q.*, et al.* (2011). Systematic discovery of Rab GTPases with synaptic functions in Drosophila. Curr Biol *21*, 1704-1715.

Charrasse, S., Comunale, F., De Rossi, S., Echard, A., and Gauthier-Rouviere, C. (2013). Rab35 regulates cadherin-mediated adherens junction formation and myoblast fusion. Mol Biol Cell *24*, 234-245.

Chen, L., Hu, J., Yun, Y., and Wang, T. (2010). Rab36 regulates the spatial distribution of late endosomes and lysosomes through a similar mechanism to Rab34. Mol Membr Biol *27*, 23-30.

Chen, S., Liang, M.C., Chia, J.N., Ngsee, J.K., and Ting, A.E. (2001). Rab8b and its interacting partner TRIP8b are involved in regulated secretion in AtT20 cells. J Biol Chem *276*, 13209-13216.

Chen, S.H., Chen, S., Tokarev, A.A., Liu, F., Jedd, G., and Segev, N. (2005). Ypt31/32 GTPases and their novel F-box effector protein Rcy1 regulate protein recycling. Mol Biol Cell *16*, 178-192.

Chen, Y., Samaraweera, P., Sun, T.T., Kreibich, G., and Orlow, S.J. (2002). Rab27b association with melanosomes: dominant negative mutants disrupt melanosomal movement. J Invest Dermatol *118*, 933-940.

Cherry, S., Jin, E.J., Ozel, M.N., Lu, Z., Agi, E., Wang, D., Jung, W.H., Epstein, D., Meinertzhagen, I.A., Chan, C.C.*, et al.* (2013). Charcot-Marie-Tooth 2B mutations in rab7 cause dosage-dependent neurodegeneration due to partial loss of function. Elife *2*, e01064.

Chevallier, J., Koop, C., Srivastava, A., Petrie, R.J., Lamarche-Vane, N., and Presley, J.F. (2009). Rab35 regulates neurite outgrowth and cell shape. FEBS Lett *583*, 1096-1101.

Cicek, I.O., Karaca, S., Brankatschk, M., Eaton, S., Urlaub, H., and Shcherbata, H.R. (2016). Hedgehog Signaling Strength Is Orchestrated by the mir-310 Cluster of MicroRNAs in Response to Diet. Genetics *202*, 1167-1183.

Cohen-Solal, K.A., Sood, R., Marin, Y., Crespo-Carbone, S.M., Sinsimer, D., Martino, J.J., Robbins, C., Makalowska, I., Trent, J., and Chen, S. (2003). Identification and characterization of mouse Rab32 by mRNA and protein expression analysis. Biochim Biophys Acta *1651*, 68-75.

Compagnon, J., Gervais, L., Roman, M.S., Chamot-Boeuf, S., and Guichet, A. (2009). Interplay between Rab5 and PtdIns(4,5)P2 controls early endocytosis in the Drosophila germline. J Cell Sci *122*, 25-35.

Corrigan, L., Redhai, S., Leiblich, A., Fan, S.J., Perera, S.M., Patel, R., Gandy, C., Wainwright, S.M., Morris, J.F., Hamdy, F.*, et al.* (2014). BMP-regulated exosomes from Drosophila male reproductive glands reprogram female behavior. J Cell Biol *206*, 671-688.

Cox, J.V., Kansal, R., and Whitt, M.A. (2016). Rab43 regulates the sorting of a subset of membrane protein cargo through the medial Golgi. Mol Biol Cell *27*, 1834-1844.

Cruz, F.M., Colbert, J.D., and Rock, K.L. (2020). The GTPase Rab39a promotes phagosome maturation into MHC-I antigen-presenting compartments. EMBO J *39*, e102020.

D'Agostino, L., Nie, Y., Goswami, S., Tong, K., Yu, S., Bandyopadhyay, S., Flores, J., Zhang, X., Balasubramanian, I., Joseph, I.*, et al.* (2019). Recycling Endosomes in Mature Epithelia Restrain Tumorigenic Signaling. Cancer Res *79*, 4099-4112.

Darchen, F., Zahraoui, A., Hammel, F., Monteils, M.P., Tavitian, A., and Scherman, D. (1990). Association of the GTP-binding protein Rab3A with bovine adrenal chromaffin granules. Proc Natl Acad Sci U S A *87*, 5692-5696.

de Madrid, B.H., Greenberg, L., and Hatini, V. (2015). RhoGAP68F controls transport of adhesion proteins in Rab4 endosomes to modulate epithelial morphogenesis of Drosophila leg discs. Dev Biol *399*, 283-295.

Dejgaard, S.Y., Murshid, A., Erman, A., Kizilay, O., Verbich, D., Lodge, R., Dejgaard, K., Ly-Hartig, T.B., Pepperkok, R., Simpson, J.C.*, et al.* (2008). Rab18 and Rab43 have key roles in ER-Golgi trafficking. J Cell Sci *121*, 2768-2781.

Dey, S., Banker, G., and Ray, K. (2017). Anterograde Transport of Rab4-Associated Vesicles Regulates Synapse Organization in Drosophila. Cell Rep *18*, 2452-2463.

Dickinson, M.E., Flenniken, A.M., Ji, X., Teboul, L., Wong, M.D., White, J.K., Meehan, T.F., Weninger, W.J., Westerberg, H., Adissu, H.*, et al.* (2016). High-throughput discovery of novel developmental phenotypes. Nature *537*, 508-514.

Dollar, G., Struckhoff, E., Michaud, J., and Cohen, R.S. (2002). Rab11 polarization of the Drosophila oocyte: a novel link between membrane trafficking, microtubule organization, and oskar mRNA localization and translation. Development *129*, 517-526.

Dong, B., Kakihara, K., Otani, T., Wada, H., and Hayashi, S. (2013). Rab9 and retromer regulate retrograde trafficking of luminal protein required for epithelial tube length control. Nat Commun *4*, 1358.

Dong, W., He, B., Qian, H., Liu, Q., Wang, D., Li, J., Wei, Z., Wang, Z., Xu, Z., Wu, G.*, et al.* (2018). RAB26-dependent autophagy protects adherens junctional integrity in acute lung injury. Autophagy *14*, 1677-1692.

Egami, Y., and Araki, N. (2012a). Rab20 regulates phagosome maturation in RAW264 macrophages during Fc gamma receptor-mediated phagocytosis. PLoS One *7*, e35663.

Egami, Y., and Araki, N. (2012b). Spatiotemporal Localization of Rab20 in Live RAW264 Macrophages during Macropinocytosis. Acta Histochem Cytochem *45*, 317-323.

Egami, Y., Fukuda, M., and Araki, N. (2011). Rab35 regulates phagosome formation through recruitment of ACAP2 in macrophages during FcgammaR-mediated phagocytosis. J Cell Sci *124*, 3557-3567.

Eggenschwiler, J.T., Bulgakov, O.V., Qin, J., Li, T., and Anderson, K.V. (2006). Mouse Rab23 regulates hedgehog signaling from smoothened to Gli proteins. Dev Biol *290*, 1-12.

Eggenschwiler, J.T., Espinoza, E., and Anderson, K.V. (2001). Rab23 is an essential negative regulator of the mouse Sonic hedgehog signalling pathway. Nature *412*, 194-198.

Ehmann, N., van de Linde, S., Alon, A., Ljaschenko, D., Keung, X.Z., Holm, T., Rings, A., DiAntonio, A., Hallermann, S., Ashery, U.*, et al.* (2014). Quantitative super-resolution imaging of Bruchpilot distinguishes active zone states. Nat Commun *5*, 4650.

Entchev, E.V., Schwabedissen, A., and Gonzalez-Gaitan, M. (2000). Gradient formation of the TGF-beta homolog Dpp. Cell *103*, 981-991.

Evans, T.M., Ferguson, C., Wainwright, B.J., Parton, R.G., and Wicking, C. (2003). Rab23, a negative regulator of hedgehog signaling, localizes to the plasma membrane and the endocytic pathway. Traffic *4*, 869-884.

Fischer von Mollard, G., Mignery, G.A., Baumert, M., Perin, M.S., Hanson, T.J., Burger, P.M., Jahn, R., and Sudhof, T.C. (1990). rab3 is a small GTP-binding protein exclusively localized to synaptic vesicles. Proc Natl Acad Sci U S A *87*, 1988-1992.

Fischer von Mollard, G., Sudhof, T.C., and Jahn, R. (1991). A small GTP-binding protein dissociates from synaptic vesicles during exocytosis. Nature *349*, 79-81.

Fuchs, E., Haas, A.K., Spooner, R.A., Yoshimura, S., Lord, J.M., and Barr, F.A. (2007). Specific Rab GTPase-activating proteins define the Shiga toxin and epidermal growth factor uptake pathways. J Cell Biol *177*, 1133-1143.

Fuller, K., O'Connell, J.T., Gordon, J., Mauti, O., and Eggenschwiler, J. (2014). Rab23 regulates Nodal signaling in vertebrate left-right patterning independently of the Hedgehog pathway. Dev Biol *391*, 182-195.

Gao, Y., Wilson, G.R., Stephenson, S.E.M., Oulad-Abdelghani, M., Charlet-Berguerand, N., Bozaoglu, K., McLean, C.A., Thomas, P.Q., Finkelstein, D.I., and Lockhart, P.J. (2020). Distribution of Parkinson's disease associated RAB39B in mouse brain tissue. Mol Brain *13*, 52.

Garg, A., and Wu, L.P. (2014). Drosophila Rab14 mediates phagocytosis in the immune response to Staphylococcus aureus. Cell Microbiol *16*, 296-310.

Geppert, M., Bolshakov, V.Y., Siegelbaum, S.A., Takei, K., De Camilli, P., Hammer, R.E., and Sudhof, T.C. (1994). The role of Rab3A in neurotransmitter release. Nature *369*, 493-497.

Giaever, G., Chu, A.M., Ni, L., Connelly, C., Riles, L., Veronneau, S., Dow, S., Lucau-Danila, A., Anderson, K., Andre, B.*, et al.* (2002). Functional profiling of the Saccharomyces cerevisiae genome. Nature *418*, 387-391.

Giagtzoglou, N., Yamamoto, S., Zitserman, D., Graves, H.K., Schulze, K.L., Wang, H., Klein, H., Roegiers, F., and Bellen, H.J. (2012). dEHBP1 controls exocytosis and recycling of Delta during asymmetric divisions. J Cell Biol *196*, 65-83.

Giannandrea, M., Bianchi, V., Mignogna, M.L., Sirri, A., Carrabino, S., D'Elia, E., Vecellio, M., Russo, S., Cogliati, F., Larizza, L.*, et al.* (2010). Mutations in the small GTPase gene RAB39B are responsible for X-linked mental retardation associated with autism, epilepsy, and macrocephaly. Am J Hum Genet *86*, 185-195.

Gillingham, A.K., Sinka, R., Torres, I.L., Lilley, K.S., and Munro, S. (2014). Toward a comprehensive map of the effectors of rab GTPases. Dev Cell *31*, 358-373.

Gorvel, J.P., Chavrier, P., Zerial, M., and Gruenberg, J. (1991). rab5 controls early endosome fusion in vitro. Cell *64*, 915-925.

Goud, B., Salminen, A., Walworth, N.C., and Novick, P.J. (1988). A GTP-binding protein required for secretion rapidly associates with secretory vesicles and the plasma membrane in yeast. Cell *53*, 753-768.

Goud, B., Zahraoui, A., Tavitian, A., and Saraste, J. (1990). Small GTP-binding protein associated with Golgi cisternae. Nature *345*, 553-556.

Graf, E.R., Daniels, R.W., Burgess, R.W., Schwarz, T.L., and DiAntonio, A. (2009). Rab3 dynamically controls protein composition at active zones. Neuron *64*, 663-677.

Guerra, F., and Bucci, C. (2016). Multiple Roles of the Small GTPase Rab7. Cells *5*.

Guo, W., Roth, D., Walch-Solimena, C., and Novick, P. (1999). The exocyst is an effector for Sec4p, targeting secretory vesicles to sites of exocytosis. EMBO J *18*, 1071-1080.

Haas, A.K., Yoshimura, S., Stephens, D.J., Preisinger, C., Fuchs, E., and Barr, F.A. (2007). Analysis of GTPase-activating proteins: Rab1 and Rab43 are key Rabs required to maintain a functional Golgi complex in human cells. J Cell Sci *120*, 2997-3010.

Higashio, H., Satoh, Y., and Saino, T. (2016). Mast cell degranulation is negatively regulated by the Munc13-4-binding small-guanosine triphosphatase Rab37. Sci Rep *6*, 22539.

Huber, L.A., Pimplikar, S., Parton, R.G., Virta, H., Zerial, M., and Simons, K. (1993). Rab8, a small GTPase involved in vesicular traffic between the TGN and the basolateral plasma membrane. J Cell Biol *123*, 35-45.

Hume, A.N., Collinson, L.M., Rapak, A., Gomes, A.Q., Hopkins, C.R., and Seabra, M.C. (2001). Rab27a regulates the peripheral distribution of melanosomes in melanocytes. J Cell Biol *152*, 795-808.

Hunziker, W., and Peters, P.J. (1998). Rab17 localizes to recycling endosomes and regulates receptor-mediated transcytosis in epithelial cells. J Biol Chem *273*, 15734-15741.

Isabella, A.J., and Horne-Badovinac, S. (2016). Rab10-Mediated Secretion Synergizes with Tissue Movement to Build a Polarized Basement Membrane Architecture for Organ Morphogenesis. Dev Cell *38*, 47-60.

Ishida, M., Ohbayashi, N., Maruta, Y., Ebata, Y., and Fukuda, M. (2012). Functional involvement of Rab1A in microtubule-dependent anterograde melanosome transport in melanocytes. J Cell Sci *125*, 5177-5187.

Itoh, T., Fujita, N., Kanno, E., Yamamoto, A., Yoshimori, T., and Fukuda, M. (2008). Golgi-resident small GTPase Rab33B interacts with Atg16L and modulates autophagosome formation. Mol Biol Cell *19*, 2916-2925.

Iwanami, N., Nakamura, Y., Satoh, T., Liu, Z., and Satoh, A.K. (2016). Rab6 Is Required for Multiple Apical Transport Pathways but Not the Basolateral Transport Pathway in Drosophila Photoreceptors. PLoS Genet *12*, e1005828.

Jacob, A., Jing, J., Lee, J., Schedin, P., Gilbert, S.M., Peden, A.A., Junutula, J.R., and Prekeris, R. (2013). Rab40b regulates trafficking of MMP2 and MMP9 during invadopodia formation and invasion of breast cancer cells. J Cell Sci *126*, 4647-4658.

Jean, S., Cox, S., Nassari, S., and Kiger, A.A. (2015). Starvation-induced MTMR13 and RAB21 activity regulates VAMP8 to promote autophagosome-lysosome fusion. EMBO Rep *16*, 297-311.

Jean, S., Cox, S., Schmidt, E.J., Robinson, F.L., and Kiger, A. (2012). Sbf/MTMR13 coordinates PI(3)P and Rab21 regulation in endocytic control of cellular remodeling. Mol Biol Cell *23*, 2723-2740.

Jedd, G., Mulholland, J., and Segev, N. (1997). Two new Ypt GTPases are required for exit from the yeast trans-Golgi compartment. J Cell Biol *137*, 563-580.

Jedd, G., Richardson, C., Litt, R., and Segev, N. (1995). The Ypt1 GTPase is essential for the first two steps of the yeast secretory pathway. J Cell Biol *131*, 583-590.

Jewett, C.E., Vanderleest, T.E., Miao, H., Xie, Y., Madhu, R., Loerke, D., and Blankenship, J.T. (2017). Planar polarized Rab35 functions as an oscillatory ratchet during cell intercalation in the Drosophila epithelium. Nat Commun *8*, 476.

Jin, E.J., Chan, C.C., Agi, E., Cherry, S., Hanacik, E., Buszczak, M., and Hiesinger, P.R. (2012). Similarities of Drosophila rab GTPases based on expression profiling: completion and analysis of the rab-Gal4 kit. PLoS One *7*, e40912.

Jin, R.U., and Mills, J.C. (2014). RAB26 coordinates lysosome traffic and mitochondrial localization. J Cell Sci *127*, 1018-1032.

Junutula, J.R., De Maziere, A.M., Peden, A.A., Ervin, K.E., Advani, R.J., van Dijk, S.M., Klumperman, J., and Scheller, R.H. (2004). Rab14 is involved in membrane trafficking between the Golgi complex and endosomes. Mol Biol Cell *15*, 2218-2229.

Kadowaki, T., Yamaguchi, Y., Kido, M.A., Abe, T., Ogawa, K., Tokuhisa, M., Gao, W., Okamoto, K., Kiyonari, H., and Tsukuba, T. (2020). The large GTPase Rab44 regulates granule exocytosis in mast cells and IgE-mediated anaphylaxis. Cell Mol Immunol *17*, 1287-1289.

Kauppi, M., Simonsen, A., Bremnes, B., Vieira, A., Callaghan, J., Stenmark, H., and Olkkonen, V.M. (2002). The small GTPase Rab22 interacts with EEA1 and controls endosomal membrane trafficking. J Cell Sci *115*, 899-911.

Kawamura, N., Sun-Wada, G.H., Aoyama, M., Harada, A., Takasuga, S., Sasaki, T., and Wada, Y. (2012). Delivery of endosomes to lysosomes via microautophagy in the visceral endoderm of mouse embryos. Nat Commun *3*, 1071.

Kawamura, S., Nagano, M., Toshima, J.Y., and Toshima, J. (2014). Analysis of subcellular localization and function of the yeast Rab6 homologue, Ypt6p, using a novel amino-terminal tagging strategy. Biochem Biophys Res Commun *450*, 519-525.

Ke, H., Feng, Z., Liu, M., Sun, T., Dai, J., Ma, M., Liu, L.P., Ni, J.Q., and Pastor-Pareja, J.C. (2018). Collagen secretion screening in Drosophila supports a common secretory machinery and multiple Rab requirements. J Genet Genomics.

Kelly, E.E., Giordano, F., Horgan, C.P., Jollivet, F., Raposo, G., and McCaffrey, M.W. (2012). Rab30 is required for the morphological integrity of the Golgi apparatus. Biol Cell *104*, 84-101.

Kirisako, T., Baba, M., Ishihara, N., Miyazawa, K., Ohsumi, M., Yoshimori, T., Noda, T., and Ohsumi, Y. (1999). Formation process of autophagosome is traced with Apg8/Aut7p in yeast. J Cell Biol *147*, 435-446.

Kohler, K., Louvard, D., and Zahraoui, A. (2004). Rab13 regulates PKA signaling during tight junction assembly. J Cell Biol *165*, 175-180.

Kouranti, I., Sachse, M., Arouche, N., Goud, B., and Echard, A. (2006). Rab35 regulates an endocytic recycling pathway essential for the terminal steps of cytokinesis. Curr Biol *16*, 1719-1725.

Kretzer, N.M., Theisen, D.J., Tussiwand, R., Briseno, C.G., Grajales-Reyes, G.E., Wu, X., Durai, V., Albring, J., Bagadia, P., Murphy, T.L.*, et al.* (2016). RAB43 facilitates cross-presentation of cell-associated antigens by CD8alpha+ dendritic cells. J Exp Med *213*, 2871-2883.

Kuwahara, T., Inoue, K., D'Agati, V.D., Fujimoto, T., Eguchi, T., Saha, S., Wolozin, B., Iwatsubo, T., and Abeliovich, A. (2016). LRRK2 and RAB7L1 coordinately regulate axonal morphology and lysosome integrity in diverse cellular contexts. Sci Rep *6*, 29945.

Laiouar, S., Berns, N., Brech, A., and Riechmann, V. (2020). RabX1 Organizes a Late Endosomal Compartment that Forms Tubular Connections to Lysosomes Consistent with a "Kiss and Run" Mechanism. Curr Biol *30*, 1177-1188 e1175.

Larance, M., Ramm, G., Stockli, J., van Dam, E.M., Winata, S., Wasinger, V., Simpson, F., Graham, M., Junutula, J.R., Guilhaus, M.*, et al.* (2005). Characterization of the role of the Rab GTPase-activating protein AS160 in insulin-regulated GLUT4 trafficking. J Biol Chem *280*, 37803-37813.

Lerner, D.W., McCoy, D., Isabella, A.J., Mahowald, A.P., Gerlach, G.F., Chaudhry, T.A., and Horne-Badovinac, S. (2013). A Rab10-dependent mechanism for polarized basement membrane secretion during organ morphogenesis. Dev Cell *24*, 159-168.

Lipatova, Z., Belogortseva, N., Zhang, X.Q., Kim, J., Taussig, D., and Segev, N. (2012). Regulation of selective autophagy onset by a Ypt/Rab GTPase module. Proc Natl Acad Sci U S A *109*, 6981-6986.

Liu, S., Hunt, L., and Storrie, B. (2013). Rab41 is a novel regulator of Golgi apparatus organization that is needed for ER-to-Golgi trafficking and cell growth. PLoS One *8*, e71886.

Liu, S., Majeed, W., Kudlyk, T., Lupashin, V., and Storrie, B. (2016). Identification of Rab41/6d Effectors Provides an Explanation for the Differential Effects of Rab41/6d and Rab6a/a' on Golgi Organization. Front Cell Dev Biol *4*, 13.

Ljubicic, S., Bezzi, P., Brajkovic, S., Nesca, V., Guay, C., Ohbayashi, N., Fukuda, M., Abderrhamani, A., and Regazzi, R. (2013). The GTPase Rab37 Participates in the Control of Insulin Exocytosis. PLoS One *8*, e68255.

Lombardi, D., Soldati, T., Riederer, M.A., Goda, Y., Zerial, M., and Pfeffer, S.R. (1993). Rab9 functions in transport between late endosomes and the trans Golgi network. EMBO J *12*, 677-682.

Lorincz, P., Toth, S., Benko, P., Lakatos, Z., Boda, A., Glatz, G., Zobel, M., Bisi, S., Hegedus, K., Takats, S.*, et al.* (2017). Rab2 promotes autophagic and endocytic lysosomal degradation. J Cell Biol *216*, 1937-1947.

Lund, V.K., Madsen, K.L., and Kjaerulff, O. (2018). Drosophila Rab2 controls endosome-lysosome fusion and LAMP delivery to late endosomes. Autophagy *14*, 1520-1542.

Luo, Z., and Gallwitz, D. (2003). Biochemical and genetic evidence for the involvement of yeast Ypt6-GTPase in protein retrieval to different Golgi compartments. J Biol Chem *278*, 791-799.

Lutcke, A., Jansson, S., Parton, R.G., Chavrier, P., Valencia, A., Huber, L.A., Lehtonen, E., and Zerial, M. (1993). Rab17, a novel small GTPase, is specific for epithelial cells and is induced during cell polarization. J Cell Biol *121*, 553-564.

Lv, P., Sheng, Y., Zhao, Z., Zhao, W., Gu, L., Xu, T., and Song, E. (2015). Targeted disruption of Rab10 causes early embryonic lethality. Protein Cell *6*, 463-467.

Ma, J., Plesken, H., Treisman, J.E., Edelman-Novemsky, I., and Ren, M. (2004). Lightoid and Claret: a rab GTPase and its putative guanine nucleotide exchange factor in biogenesis of Drosophila eye pigment granules. Proc Natl Acad Sci U S A *101*, 11652-11657.

MacLeod, D.A., Rhinn, H., Kuwahara, T., Zolin, A., Di Paolo, G., McCabe, B.D., Marder, K.S., Honig, L.S., Clark, L.N., Small, S.A.*, et al.* (2013). RAB7L1 interacts with LRRK2 to modify intraneuronal protein sorting and Parkinson's disease risk. Neuron *77*, 425-439.

Mallard, F., Tang, B.L., Galli, T., Tenza, D., Saint-Pol, A., Yue, X., Antony, C., Hong, W., Goud, B., and Johannes, L. (2002). Early/recycling endosomes-to-TGN transport involves two SNARE complexes and a Rab6 isoform. J Cell Biol *156*, 653-664.

Masuda, E.S., Luo, Y., Young, C., Shen, M., Rossi, A.B., Huang, B.C., Yu, S., Bennett, M.K., Payan, D.G., and Scheller, R.H. (2000). Rab37 is a novel mast cell specific GTPase localized to secretory granules. FEBS Lett *470*, 61-64.

Matsui, T., Itoh, T., and Fukuda, M. (2011). Small GTPase Rab12 regulates constitutive degradation of transferrin receptor. Traffic *12*, 1432-1443.

Matsui, T., Ohbayashi, N., and Fukuda, M. (2012). The Rab interacting lysosomal protein (RILP) homology domain functions as a novel effector domain for small GTPase Rab36: Rab36 regulates retrograde melanosome transport in melanocytes. J Biol Chem *287*, 28619-28631.

Mavor, L.M., Miao, H., Zuo, Z., Holly, R.M., Xie, Y., Loerke, D., and Blankenship, J.T. (2016). Rab8 directs furrow ingression and membrane addition during epithelial formation in Drosophila melanogaster. Development *143*, 892-903.

Meresse, S., Gorvel, J.P., and Chavrier, P. (1995). The rab7 GTPase resides on a vesicular compartment connected to lysosomes. J Cell Sci *108 ( Pt 11)*, 3349-3358.

Mignogna, M.L., Giannandrea, M., Gurgone, A., Fanelli, F., Raimondi, F., Mapelli, L., Bassani, S., Fang, H., Van Anken, E., Alessio, M.*, et al.* (2015). The intellectual disability protein RAB39B selectively regulates GluA2 trafficking to determine synaptic AMPAR composition. Nat Commun *6*, 6504.

Nagy, P., Szatmari, Z., Sandor, G.O., Lippai, M., Hegedus, K., and Juhasz, G. (2017). Drosophila Atg16 promotes enteroendocrine cell differentiation via regulation of intestinal Slit/Robo signaling. Development *144*, 3990-4001.

Nakamura, S., Takemura, T., Tan, L., Nagata, Y., Yokota, D., Hirano, I., Shigeno, K., Shibata, K., Fujie, M., Fujisawa, S.*, et al.* (2011). Small GTPase RAB45-mediated p38 activation in apoptosis of chronic myeloid leukemia progenitor cells. Carcinogenesis *32*, 1758-1772.

Nakazawa, H., Sada, T., Toriyama, M., Tago, K., Sugiura, T., Fukuda, M., and Inagaki, N. (2012). Rab33a mediates anterograde vesicular transport for membrane exocytosis and axon outgrowth. J Neurosci *32*, 12712-12725.

Nam, K.T., Lee, H.J., Smith, J.J., Lapierre, L.A., Kamath, V.P., Chen, X., Aronow, B.J., Yeatman, T.J., Bhartur, S.G., Calhoun, B.C.*, et al.* (2010). Loss of Rab25 promotes the development of intestinal neoplasia in mice and is associated with human colorectal adenocarcinomas. J Clin Invest *120*, 840-849.

Ng, E.L., Wang, Y., and Tang, B.L. (2007). Rab22B's role in trans-Golgi network membrane dynamics. Biochem Biophys Res Commun *361*, 751-757.

Nickerson, D.P., Russell, M.R.G., Lo, S.Y., Chapin, H.C., Milnes, J., and Merz, A.J. (2012). Termination of isoform-selective Vps21/Rab5 signaling at endolysosomal organelles by Msb3/Gyp3. Traffic *13*, 1411-1428.

Nokes, R.L., Fields, I.C., Collins, R.N., and Folsch, H. (2008). Rab13 regulates membrane trafficking between TGN and recycling endosomes in polarized epithelial cells. J Cell Biol *182*, 845-853.

Nyitrai, H., Wang, S.S.H., and Kaeser, P.S. (2020). ELKS1 Captures Rab6-Marked Vesicular Cargo in Presynaptic Nerve Terminals. Cell Rep *31*, 107712.

Olkkonen, V.M., Dupree, P., Killisch, I., Lutcke, A., Zerial, M., and Simons, K. (1993). Molecular cloning and subcellular localization of three GTP-binding proteins of the rab subfamily. J Cell Sci *106 ( Pt 4)*, 1249-1261.

Opdam, F.J., Echard, A., Croes, H.J., van den Hurk, J.A., van de Vorstenbosch, R.A., Ginsel, L.A., Goud, B., and Fransen, J.A. (2000). The small GTPase Rab6B, a novel Rab6 subfamily member, is cell-type specifically expressed and localised to the Golgi apparatus. J Cell Sci *113 ( Pt 15)*, 2725-2735.

Ostrowski, M., Carmo, N.B., Krumeich, S., Fanget, I., Raposo, G., Savina, A., Moita, C.F., Schauer, K., Hume, A.N., Freitas, R.P.*, et al.* (2010). Rab27a and Rab27b control different steps of the exosome secretion pathway. Nat Cell Biol *12*, 19-30; sup pp 11-13.

Ozeki, S., Cheng, J., Tauchi-Sato, K., Hatano, N., Taniguchi, H., and Fujimoto, T. (2005). Rab18 localizes to lipid droplets and induces their close apposition to the endoplasmic reticulum-derived membrane. J Cell Sci *118*, 2601-2611.

Pataki, C., Matusek, T., Kurucz, E., Ando, I., Jenny, A., and Mihaly, J. (2010). Drosophila Rab23 is involved in the regulation of the number and planar polarization of the adult cuticular hairs. Genetics *184*, 1051-1065.

Pellinen, T., Arjonen, A., Vuoriluoto, K., Kallio, K., Fransen, J.A., and Ivaska, J. (2006). Small GTPase Rab21 regulates cell adhesion and controls endosomal traffic of beta1-integrins. J Cell Biol *173*, 767-780.

Pellinen, T., Tuomi, S., Arjonen, A., Wolf, M., Edgren, H., Meyer, H., Grosse, R., Kitzing, T., Rantala, J.K., Kallioniemi, O.*, et al.* (2008). Integrin trafficking regulated by Rab21 is necessary for cytokinesis. Dev Cell *15*, 371-385.

Plutner, H., Cox, A.D., Pind, S., Khosravi-Far, R., Bourne, J.R., Schwaninger, R., Der, C.J., and Balch, W.E. (1991). Rab1b regulates vesicular transport between the endoplasmic reticulum and successive Golgi compartments. J Cell Biol *115*, 31-43.

Purcell, K., and Artavanis-Tsakonas, S. (1999). The developmental role of warthog, the notch modifier encoding Drab6. J Cell Biol *146*, 731-740.

Purlyte, E., Dhekne, H.S., Sarhan, A.R., Gomez, R., Lis, P., Wightman, M., Martinez, T.N., Tonelli, F., Pfeffer, S.R., and Alessi, D.R. (2018). Rab29 activation of the Parkinson's disease-associated LRRK2 kinase. EMBO J *37*, 1-18.

Riedel, D., Antonin, W., Fernandez-Chacon, R., Alvarez de Toledo, G., Jo, T., Geppert, M., Valentijn, J.A., Valentijn, K., Jamieson, J.D., Sudhof, T.C.*, et al.* (2002). Rab3D is not required for exocrine exocytosis but for maintenance of normally sized secretory granules. Mol Cell Biol *22*, 6487-6497.

Rodriguez-Gabin, A.G., Almazan, G., and Larocca, J.N. (2004). Vesicle transport in oligodendrocytes: probable role of Rab40c protein. J Neurosci Res *76*, 758-770.

Rodriguez-Gabin, A.G., Cammer, M., Almazan, G., Charron, M., and Larocca, J.N. (2001). Role of rRAB22b, an oligodendrocyte protein, in regulation of transport of vesicles from trans Golgi to endocytic compartments. J Neurosci Res *66*, 1149-1160.

Rodriguez-Gabin, A.G., Yin, X., Si, Q., and Larocca, J.N. (2009). Transport of mannose-6-phosphate receptors from the trans-Golgi network to endosomes requires Rab31. Exp Cell Res *315*, 2215-2230.

Roosing, S., Rohrschneider, K., Beryozkin, A., Sharon, D., Weisschuh, N., Staller, J., Kohl, S., Zelinger, L., Peters, T.A., Neveling, K.*, et al.* (2013). Mutations in RAB28, encoding a farnesylated small GTPase, are associated with autosomal-recessive cone-rod dystrophy. Am J Hum Genet *93*, 110-117.

Saito-Ohara, F., Fukuda, Y., Ito, M., Agarwala, K.L., Hayashi, M., Matsuo, M., Imoto, I., Yamakawa, K., Nakamura, Y., and Inazawa, J. (2002). The Xq22 inversion breakpoint interrupted a novel Ras-like GTPase gene in a patient with Duchenne muscular dystrophy and profound mental retardation. Am J Hum Genet *71*, 637-645.

Salminen, A., and Novick, P.J. (1987). A ras-like protein is required for a post-Golgi event in yeast secretion. Cell *49*, 527-538.

Sano, H., Eguez, L., Teruel, M.N., Fukuda, M., Chuang, T.D., Chavez, J.A., Lienhard, G.E., and McGraw, T.E. (2007). Rab10, a target of the AS160 Rab GAP, is required for insulin-stimulated translocation of GLUT4 to the adipocyte plasma membrane. Cell Metab *5*, 293-303.

Sato, T., Iwano, T., Kunii, M., Matsuda, S., Mizuguchi, R., Jung, Y., Hagiwara, H., Yoshihara, Y., Yuzaki, M., Harada, R.*, et al.* (2014). Rab8a and Rab8b are essential for several apical transport pathways but insufficient for ciliogenesis. J Cell Sci *127*, 422-431.

Sato, T., Mushiake, S., Kato, Y., Sato, K., Sato, M., Takeda, N., Ozono, K., Miki, K., Kubo, Y., Tsuji, A.*, et al.* (2007). The Rab8 GTPase regulates apical protein localization in intestinal cells. Nature *448*, 366-369.

Schimmoller, F., and Riezman, H. (1993). Involvement of Ypt7p, a small GTPase, in traffic from late endosome to the vacuole in yeast. J Cell Sci *106 ( Pt 3)*, 823-830.

Schluter, O.M., Khvotchev, M., Jahn, R., and Sudhof, T.C. (2002). Localization versus function of Rab3 proteins. Evidence for a common regulatory role in controlling fusion. J Biol Chem *277*, 40919-40929.

Schluter, O.M., Schmitz, F., Jahn, R., Rosenmund, C., and Sudhof, T.C. (2004). A complete genetic analysis of neuronal Rab3 function. J Neurosci *24*, 6629-6637.

Seachrist, J.L., Anborgh, P.H., and Ferguson, S.S. (2000). beta 2-adrenergic receptor internalization, endosomal sorting, and plasma membrane recycling are regulated by rab GTPases. J Biol Chem *275*, 27221-27228.

Sechi, S., Frappaolo, A., Fraschini, R., Capalbo, L., Gottardo, M., Belloni, G., Glover, D.M., Wainman, A., and Giansanti, M.G. (2017). Rab1 interacts with GOLPH3 and controls Golgi structure and contractile ring constriction during cytokinesis in Drosophila melanogaster. Open Biol *7*.

Segev, N., Mulholland, J., and Botstein, D. (1988). The yeast GTP-binding YPT1 protein and a mammalian counterpart are associated with the secretion machinery. Cell *52*, 915-924.

Seto, S., Sugaya, K., Tsujimura, K., Nagata, T., Horii, T., and Koide, Y. (2013). Rab39a interacts with phosphatidylinositol 3-kinase and negatively regulates autophagy induced by lipopolysaccharide stimulation in macrophages. PLoS One *8*, e83324.

Seto, S., Tsujimura, K., and Koide, Y. (2011). Rab GTPases regulating phagosome maturation are differentially recruited to mycobacterial phagosomes. Traffic *12*, 407-420.

Shim, J., Lee, S.M., Lee, M.S., Yoon, J., Kweon, H.S., and Kim, Y.J. (2010). Rab35 mediates transport of Cdc42 and Rac1 to the plasma membrane during phagocytosis. Mol Cell Biol *30*, 1421-1433.

Shintani, M., Tada, M., Kobayashi, T., Kajiho, H., Kontani, K., and Katada, T. (2007). Characterization of Rab45/RASEF containing EF-hand domain and a coiled-coil motif as a self-associating GTPase. Biochem Biophys Res Commun *357*, 661-667.

Simpson, J.C., Griffiths, G., Wessling-Resnick, M., Fransen, J.A., Bennett, H., and Jones, A.T. (2004). A role for the small GTPase Rab21 in the early endocytic pathway. J Cell Sci *117*, 6297-6311.

Singer-Kruger, B., Stenmark, H., Dusterhoft, A., Philippsen, P., Yoo, J.S., Gallwitz, D., and Zerial, M. (1994). Role of three rab5-like GTPases, Ypt51p, Ypt52p, and Ypt53p, in the endocytic and vacuolar protein sorting pathways of yeast. J Cell Biol *125*, 283-298.

Sinka, R., Gillingham, A.K., Kondylis, V., and Munro, S. (2008). Golgi coiled-coil proteins contain multiple binding sites for Rab family G proteins. J Cell Biol *183*, 607-615.

Speight, P., and Silverman, M. (2005). Diacylglycerol-activated Hmunc13 serves as an effector of the GTPase Rab34. Traffic *6*, 858-865.

Suda, Y., Kurokawa, K., Hirata, R., and Nakano, A. (2013). Rab GAP cascade regulates dynamics of Ypt6 in the Golgi traffic. Proc Natl Acad Sci U S A *110*, 18976-18981.

Sun, P., Yamamoto, H., Suetsugu, S., Miki, H., Takenawa, T., and Endo, T. (2003). Small GTPase Rah/Rab34 is associated with membrane ruffles and macropinosomes and promotes macropinosome formation. J Biol Chem *278*, 4063-4071.

Sun, Y., Shestakova, A., Hunt, L., Sehgal, S., Lupashin, V., and Storrie, B. (2007). Rab6 regulates both ZW10/RINT-1 and conserved oligomeric Golgi complex-dependent Golgi trafficking and homeostasis. Mol Biol Cell *18*, 4129-4142.

Tan, R., Wang, W., Wang, S., Wang, Z., Sun, L., He, W., Fan, R., Zhou, Y., Xu, X., Hong, W.*, et al.* (2013). Small GTPase Rab40c associates with lipid droplets and modulates the biogenesis of lipid droplets. PLoS One *8*, e63213.

Thibault, S.T., Singer, M.A., Miyazaki, W.Y., Milash, B., Dompe, N.A., Singh, C.M., Buchholz, R., Demsky, M., Fawcett, R., Francis-Lang, H.L.*, et al.* (2004). A complementary transposon tool kit for Drosophila melanogaster using P and piggyBac. Nat Genet *36*, 283-287.

Thomas, C., Rousset, R., and Noselli, S. (2009). JNK signalling influences intracellular trafficking during Drosophila morphogenesis through regulation of the novel target gene Rab30. Dev Biol *331*, 250-260.

Tisdale, E.J., and Balch, W.E. (1996). Rab2 is essential for the maturation of pre-Golgi intermediates. J Biol Chem *271*, 29372-29379.

Tisdale, E.J., Bourne, J.R., Khosravi-Far, R., Der, C.J., and Balch, W.E. (1992). GTP-binding mutants of rab1 and rab2 are potent inhibitors of vesicular transport from the endoplasmic reticulum to the Golgi complex. J Cell Biol *119*, 749-761.

Tokuhisa, M., Kadowaki, T., Ogawa, K., Yamaguchi, Y., Kido, M.A., Gao, W., Umeda, M., and Tsukuba, T. (2020). Expression and localisation of Rab44 in immune-related cells change during cell differentiation and stimulation. Sci Rep *10*, 10728.

Tolmachova, T., Abrink, M., Futter, C.E., Authi, K.S., and Seabra, M.C. (2007). Rab27b regulates number and secretion of platelet dense granules. Proc Natl Acad Sci U S A *104*, 5872-5877.

Tong, C., Ohyama, T., Tien, A.C., Rajan, A., Haueter, C.M., and Bellen, H.J. (2011). Rich regulates target specificity of photoreceptor cells and N-cadherin trafficking in the Drosophila visual system via Rab6. Neuron *71*, 447-459.

Trischler, M., Stoorvogel, W., and Ullrich, O. (1999). Biochemical analysis of distinct Rab5- and Rab11-positive endosomes along the transferrin pathway. J Cell Sci *112 ( Pt 24)*, 4773-4783.

Tuvim, M.J., Adachi, R., Chocano, J.F., Moore, R.H., Lampert, R.M., Zera, E., Romero, E., Knoll, B.J., and Dickey, B.F. (1999). Rab3D, a small GTPase, is localized on mast cell secretory granules and translocates to the plasma membrane upon exocytosis. Am J Respir Cell Mol Biol *20*, 79-89.

Ullrich, O., Reinsch, S., Urbe, S., Zerial, M., and Parton, R.G. (1996). Rab11 regulates recycling through the pericentriolar recycling endosome. J Cell Biol *135*, 913-924.

Urbe, S., Huber, L.A., Zerial, M., Tooze, S.A., and Parton, R.G. (1993). Rab11, a small GTPase associated with both constitutive and regulated secretory pathways in PC12 cells. FEBS Lett *334*, 175-182.

Uytterhoeven, V., Kuenen, S., Kasprowicz, J., Miskiewicz, K., and Verstreken, P. (2011). Loss of skywalker reveals synaptic endosomes as sorting stations for synaptic vesicle proteins. Cell *145*, 117-132.

Valsdottir, R., Hashimoto, H., Ashman, K., Koda, T., Storrie, B., and Nilsson, T. (2001). Identification of rabaptin-5, rabex-5, and GM130 as putative effectors of rab33b, a regulator of retrograde traffic between the Golgi apparatus and ER. FEBS Lett *508*, 201-209.

van der Sluijs, P., Hull, M., Webster, P., Male, P., Goud, B., and Mellman, I. (1992). The small GTP-binding protein rab4 controls an early sorting event on the endocytic pathway. Cell *70*, 729-740.

Vitelli, R., Santillo, M., Lattero, D., Chiariello, M., Bifulco, M., Bruni, C.B., and Bucci, C. (1997). Role of the small GTPase Rab7 in the late endocytic pathway. J Biol Chem *272*, 4391-4397.

Wang, C., Liu, Z., and Huang, X. (2012). Rab32 is important for autophagy and lipid storage in Drosophila. PLoS One *7*, e32086.

Wang, S., Ma, Z., Xu, X., Wang, Z., Sun, L., Zhou, Y., Lin, X., Hong, W., and Wang, T. (2014). A role of Rab29 in the integrity of the trans-Golgi network and retrograde trafficking of mannose-6-phosphate receptor. PLoS One *9*, e96242.

Wang, T., and Hong, W. (2002). Interorganellar regulation of lysosome positioning by the Golgi apparatus through Rab34 interaction with Rab-interacting lysosomal protein. Mol Biol Cell *13*, 4317-4332.

Wasmeier, C., Romao, M., Plowright, L., Bennett, D.C., Raposo, G., and Seabra, M.C. (2006). Rab38 and Rab32 control post-Golgi trafficking of melanogenic enzymes. J Cell Biol *175*, 271-281.

West, R.J., Lu, Y., Marie, B., Gao, F.B., and Sweeney, S.T. (2015). Rab8, POSH, and TAK1 regulate synaptic growth in a Drosophila model of frontotemporal dementia. J Cell Biol *208*, 931-947.

Wilson, S.M., Yip, R., Swing, D.A., O'Sullivan, T.N., Zhang, Y., Novak, E.K., Swank, R.T., Russell, L.B., Copeland, N.G., and Jenkins, N.A. (2000). A mutation in Rab27a causes the vesicle transport defects observed in ashen mice. Proc Natl Acad Sci U S A *97*, 7933-7938.

Woichansky, I., Beretta, C.A., Berns, N., and Riechmann, V. (2016). Three mechanisms control E-cadherin localization to the zonula adherens. Nat Commun *7*, 10834.

Wucherpfennig, T., Wilsch-Brauninger, M., and Gonzalez-Gaitan, M. (2003). Role of Drosophila Rab5 during endosomal trafficking at the synapse and evoked neurotransmitter release. J Cell Biol *161*, 609-624.

Xu, S., Liu, Y., Meng, Q., and Wang, B. (2018). Rab34 small GTPase is required for Hedgehog signaling and an early step of ciliary vesicle formation in mouse. J Cell Sci *131*.

Yamaguchi, Y., Sakai, E., Okamoto, K., Kajiya, H., Okabe, K., Naito, M., Kadowaki, T., and Tsukuba, T. (2018). Rab44, a novel large Rab GTPase, negatively regulates osteoclast differentiation by modulating intracellular calcium levels followed by NFATc1 activation. Cell Mol Life Sci *75*, 33-48.

Yang, S., and Rosenwald, A.G. (2016). Autophagy in Saccharomyces cerevisiae requires the monomeric GTP-binding proteins, Arl1 and Ypt6. Autophagy *12*, 1721-1737.

Ying, G., Boldt, K., Ueffing, M., Gerstner, C.D., Frederick, J.M., and Baehr, W. (2018). The small GTPase RAB28 is required for phagocytosis of cone outer segments by the murine retinal pigmented epithelium. J Biol Chem *293*, 17546-17558.

Yla-Anttila, P., Mikkonen, E., Happonen, K.E., Holland, P., Ueno, T., Simonsen, A., and Eskelinen, E.L. (2015). RAB24 facilitates clearance of autophagic compartments during basal conditions. Autophagy *11*, 1833-1848.

Yoshie, S., Imai, A., Nashida, T., and Shimomura, H. (2000). Expression, characterization, and localization of Rab26, a low molecular weight GTP-binding protein, in the rat parotid gland. Histochem Cell Biol *113*, 259-263.

Yoshimura, S., Egerer, J., Fuchs, E., Haas, A.K., and Barr, F.A. (2007). Functional dissection of Rab GTPases involved in primary cilium formation. J Cell Biol *178*, 363-369.

Young, J., Menetrey, J., and Goud, B. (2010). RAB6C is a retrogene that encodes a centrosomal protein involved in cell cycle progression. J Mol Biol *397*, 69-88.

Young, J., Stauber, T., del Nery, E., Vernos, I., Pepperkok, R., and Nilsson, T. (2005). Regulation of microtubule-dependent recycling at the trans-Golgi network by Rab6A and Rab6A'. Mol Biol Cell *16*, 162-177.

Yousefian, J., Troost, T., Grawe, F., Sasamura, T., Fortini, M., and Klein, T. (2013). Dmon1 controls recruitment of Rab7 to maturing endosomes in Drosophila. J Cell Sci *126*, 1583-1594.

Yu, S., Yehia, G., Wang, J., Stypulkowski, E., Sakamori, R., Jiang, P., Hernandez-Enriquez, B., Tran, T.S., Bonder, E.M., Guo, W.*, et al.* (2014). Global ablation of the mouse Rab11a gene impairs early embryogenesis and matrix metalloproteinase secretion. J Biol Chem *289*, 32030-32043.

Zacchi, P., Stenmark, H., Parton, R.G., Orioli, D., Lim, F., Giner, A., Mellman, I., Zerial, M., and Murphy, C. (1998). Rab17 regulates membrane trafficking through apical recycling endosomes in polarized epithelial cells. J Cell Biol *140*, 1039-1053.

Zahraoui, A., Joberty, G., Arpin, M., Fontaine, J.J., Hellio, R., Tavitian, A., and Louvard, D. (1994). A small rab GTPase is distributed in cytoplasmic vesicles in non polarized cells but colocalizes with the tight junction marker ZO-1 in polarized epithelial cells. J Cell Biol *124*, 101-115.

Zhang, J., Fonovic, M., Suyama, K., Bogyo, M., and Scott, M.P. (2009). Rab35 controls actin bundling by recruiting fascin as an effector protein. Science *325*, 1250-1254.

Zheng, J.Y., Koda, T., Fujiwara, T., Kishi, M., Ikehara, Y., and Kakinuma, M. (1998). A novel Rab GTPase, Rab33B, is ubiquitously expressed and localized to the medial Golgi cisternae. J Cell Sci *111 ( Pt 8)*, 1061-1069.

Zuk, P.A., and Elferink, L.A. (1999). Rab15 mediates an early endocytic event in Chinese hamster ovary cells. J Biol Chem *274*, 22303-22312.

Zuk, P.A., and Elferink, L.A. (2000). Rab15 differentially regulates early endocytic trafficking. J Biol Chem *275*, 26754-26764.
