# Supplementary material for: Systematic functional analysis of rab GTPases reveals limits of neuronal robustness to environmental challenges in flies
Source: eLife. 2021 Mar 5;10:e59594. doi: 10.7554/eLife.59594 (PMC8016483; doi:10.7554/eLife.59594)
Supplement: Supplementary file 4. — (A) Summary of developmental time for wild type and all fertile, homozygous viable rab mutants at 18°C, 25°C, and 29°C. Listed are number of days (after 24 hr of egg collection) until first 1st instar larvae, pupae, or adults appear, as well as total number of adults hatched and number of adults per vial. Days are given in mean ± SEM. (B) Summary of developmental time for wild type and tested backcrossed rab mutants at 18°C, 25°C, and 29°C. Listed are number of days (after 24 hr of egg collection) until first 1st instar larvae, pupae, or adults appear, as well as total number of adults hatched and number of adults per vial. Days are given in mean ± SEM. (C) Summary of developmental time for wild type and tested rab mutants over deficiencies at 18°C, 25°C and 29°C. Listed are number of days (after 24 hr of egg collection) until first 1st instar larvae, pupae, or adults appear, as well as total number of adults hatched and number of adults per vial. Days are given in mean ± SEM. [file elife-59594-supp4.docx]

**Supplementary Table / Supplementary File 4**

**A: Homozygous mutants**

| **18 degree** | days until… (after egg collection) | | | number of adults in total | Ø number of adults per vial |
| --- | --- | --- | --- | --- | --- |
|  | first instar larva | pupa | adult |  |  |
| control | 0 | 8.43 ± 0.13 | 16.8 ± 0.14 | 170 | 8.1 ± 1.82 |
| *rab3* | 0.4 ± 0.13 | 9.29 ± 0.18 | 18.1 ± 0.24 | 159 | 7.57 ± 1.02 |
| *rab4* | 0.1 ± 0.07 | 9.19 ± 0.09 | 17.38 ± 0.11 | 516 | 24.57 ± 2.1 |
| *rab9* | 0.29 ± 0.1 | 7.86 ± 0.08 | 16.71 ± 0.12 | 344 | 16.38 ± 1.41 |
| *rab14* | 0.1 ± 0.07 | 7.57 ± 0.11 | 15.76 ± 0.1 | 473 | 22.52 ± 1.82 |
| *rab18* | 0.33 ± 0.13 | 9.05 ± 0.21 | 17.62 ± 0.25 | 203 | 9.67 ± 1.17 |
| *rab19* | 0.1 ± 0.07 | 8.76 ± 0.14 | 18.1 ± 0.17 | 275 | 13.1 ± 1.44 |
| *rab21* | 0.29 ± 0.12 | 8.29 ± 0.1 | 16.95 ± 0.16 | 368 | 17.52 ± 0.98 |
| *rab23* | 0.24 ± 0.1 | 8.62 ± 0.11 | 17.19 ± 0.11 | 239 | 11.38 ± 1.01 |
| *rab26* | 0.24 ± 0.1 | 8.71 ± 0.1 | 17 ± 0.1 | 442 | 21.05 ± 1.91 |
| *rab27* | 0.1 ± 0.07 | 8.86 ± 0.1 | 17.1 ± 0.07 | 368 | 17.52 ± 1.16 |
| *rab32* | 0.1 ± 0.07 | 9.38 ± 0.11 | 18 ± 0.17 | 316 | 15.05 ± 0.87 |
| *rab39* | 0 | 9.05 ± 0.13 | 17.76 ± 0.1 | 523 | 24.9 ± 1.75 |
| *rab40* | 0.33 ± 0.16 | 10.19 ± 0.09 | 18.67 ± 0.14 | 261 | 12.42 ± 0.98 |
| *rabX1* | 1.71 ± 0.46 | 10.56 ± 0.26 | 19.24 ± 0.2 | 37 | 1.76 ± 0.32 |
| *rabX4* | 0.9 ± 0.14 | 12 ± 0.3 | 21.4 ± 0.53 | 8 | 0.38 ± 0.13 |
| *rabX6* | 0 | 9.14 ± 0.14 | 17.8 ± 0.13 | 446 | 21.24 ± 2.64 |

| **25 degree** | days until… (after egg collection) | | | number of adults in total | Ø number of adults per vial |
| --- | --- | --- | --- | --- | --- |
|  | first instar larva | pupa | adult |  |  |
| control | 0.15 ± 0.08 | 4.86 ± 0.08 | 8.95 ± 0.05 | 230 | 10.95 ± 2.01 |
| *rab3* | 0.2 ± 0.09 | 5 ± 0.12 | 9.2 ± 0.12 | 197 | 9.85 ± 1.45 |
| *rab4* | 0.1 ± 0.07 | 5.29 ± 0.1 | 9.25 ± 0.16 | 634 | 30.19 ± 2.06 |
| *rab9* | 0.14 ± 0.08 | 5 | 9 | 411 | 19.57 ± 1.63 |
| *rab14* | 0.1 ± 0.07 | 4.62 ± 0.11 | 8.67 ± 0.14 | 499 | 23.76 ± 1.75 |
| *rab18* | 0.05 ± 0.05 | 4.86 ± 0.08 | 8.9 ± 0.07 | 345 | 16.43 ± 1.34 |
| *rab19* | 0.24 ± 0.1 | 4.95 ± 0.08 | 9.71 ± 0.1 | 379 | 18.04 ± 1.51 |
| *rab21* | 0.19 ± 0.09 | 5 | 8.95 ± 0.05 | 412 | 19.62 ± 1.74 |
| *rab23* | 0.1 ± 0.07 | 4.86 ± 0.08 | 9 | 305 | 14.52 ± 1.71 |
| *rab26* | 0.2 ± 0.09 | 4.95 ± 0.05 | 9.1 ± 0.07 | 480 | 22.86 ± 2.1 |
| *rab27* | 0.14 ± 0.08 | 5 | 9 | 382 | 18.19 ± 1.12 |
| *rab32* | 0.29 ± 0.1 | 5.24 ±0.1 | 9.19 ± 0.11 | 459 | 21.86 ± 1.62 |
| *rab39* | 0.24 ± 0.1 | 5.14 ± 0.08 | 9.43 ± 0.11 | 627 | 29.86 ± 2.69 |
| *rab40* | 0.24 ± 0.1 | 5.7 ± 0.13 | 9.95 ± 0.15 | 241 | 11.48 ± 1.14 |
| *rabX1* | 0.56 ± 0.15 | 5.29 ± 0.14 | 9.67 ± 0.14 | 40 | 1.9 ± 0.35 |
| *rabX4* | 0.89 ± 0.08 | 7.14 ± 0.7 | 12 | 2 | 0.1 ± 0.1 |
| *rabX6* | 0.05 ± 0.05 | 5.05 ± 0.05 | 9.23 ± 0.1 | 459 | 21.86 ± 2.62 |

| **29 degree** | days until… (after egg collection) | | | # number of adults in total | Ø number of adults per vial |
| --- | --- | --- | --- | --- | --- |
|  | first instar larva | pupa | adult |  |  |
| control | 0.15 ± 0.09 | 4 | 7.67 ± 0.11 | 112 | 5.33 ± 1.21 |
| *rab3* | 0.1 ± 0.07 | 4.19 ± 0.09 | 7.57 ± 0.11 | 168 | 8 ± 0.81 |
| *rab4* | 0 | 4.52 ± 0.11 | 8 | 572 | 27 ± 2.14 |
| *rab9* | 0.14 ± 0.08 | 3.95 ± 0.05 | 7.57 ± 0.11 | 369 | 17.57 ± 1.73 |
| *rab14* | 0 | 4.05 ± 0.05 | 7.43 ± 0.11 | 516 | 24.57 ± 1.66 |
| *rab18* | 0.1 ± 0.07 | 4.19 ± 0.09 | 7.35 ± 0.11 | 291 | 13.86 ± 1.68 |
| *rab19* | 0.19 ± 0.09 | 4.05 ± 0.05 | 8.94 ± 0.18 | 132 | 7.33 ± 1.4 |
| *rab21* | 0.05 ± 0.05 | 4.19 ± 0.09 | 8 ± 0.2 | 378 | 18.9 ± 1.72 |
| *rab23* | 0.29 ± 0.1 | 4 | 7.95 ± 0.05 | 288 | 13.71 ± 0.92 |
| *rab26* | 0.29 ± 0.1 | 4.14 ± 0.08 | 7.81 ± 0.09 | 378 | 18 ± 1.76 |
| *rab27* | 0.05 ± 0.05 | 4 | 7.43 ± 0.11 | 444 | 21.14 ± 0.95 |
| *rab32* | 0.24 ± 0.1 | 4.33 ± 0.11 | 8 | 307 | 14.62 ± 1.01 |
| *rab39* | 0.19 ± 0.09 | 4.1 ± 0.07 | 7.81 ± 0.09 | 572 | 27.24 ± 2.3 |
| *rab40* | 0.33 ± 0.11 | 4 ± 0.12 | 7.31 ± 0.1 | 280 | 13.33 ± 0.74 |
| *rabX1* | 0.81 ± 0.16 | 4.75 ± 0.11 | 8.19 ± 0.1 | 38 | 1.81 ± 0.38 |
| *rabX4* | 0.75 ± 0.1 | 6.38 ± 0.56 | 13.5 ± 3.5 | 2 | 0.1 ± 0.1 |
| *rabX6* | 0.19 ± 0.09 | 4.05 ± 0.05 | 7.76 ± 0.1 | 445 | 21.19 ± 2.75 |

**B: Validation in backcrossed backgrounds**

| **18 degree** | days until… (after egg collection) | | | number of adults in total | Ø number of adults per vial |
| --- | --- | --- | --- | --- | --- |
|  | first instar larva | pupa | adult |  |  |
| control | 0.05 ± 0.05 | 8.5 ± 0.1 | 16.7 ± 0.14 | 290 | 13.81 ± 1.25 |
| *rab3­* | 0.52 ± 0.19 | 10 ± 0.41 | 18 ± 0.41 | 23 | 5.75 ± 0.48 |
| *rab4* | 0.05 ± 0.05 | 9.38 ± 0.18 | 18.52 ± 0.15 | 190 | 9.05 ± 0.79 |
| *rab9* | 0.14 ± 0.08 | 8.33 ± 0.13 | 17 ± 0.18 | 284 | 13.52 ± 0.99 |
| *rab14* | 0.1 ± 0.07 | 8.38 ± 0.11 | 16.95 ± 0.19 | 176 | 8.38 ± 0.93 |
| *rab19* | 0.05 ± 0.05 | 8.86 ± 0.2 | 18.29 ± 0.22 | 224 | 10.67 ± 1.52 |
| *rab32* | 0.33 ± 0.11 | 10.32 ± 0.17 | 18.74 ± 0.2 | 136 | 7.16 ± 1 |
| *rab39* | 0 | 8,71 ± 0.27 | 17.8 ± 0.24 | 327 | 15.57 ± 1.32 |
| *rab40* | 0.19 ± 0.09 | 9.33 ± 0.22 | 17.86 ± 0.19 | 207 | 9.86 ± 0.67 |
| *rabX1* | 0.67 ± 0.16 | 10.1 ± 0.21 | 18.38 ± 0.27 | 33 | 2.54 ± 0.37 |
| *rabX4* | 1.88 ± 0.4 | - | - | - | - |
| *rabX6* | 0 | 9.29 ± 0.14 | 18.29 ± 0.22 | 378 | 18 ± 1.62 |

­

| **25 degree** | days until… (after egg collection) | | | number of adults in total | Ø number of adults per vial |
| --- | --- | --- | --- | --- | --- |
|  | first instar larva | pupa | adult |  |  |
| control | 0.05 ± 0.05 | 5.24 ± 0.1 | 9.19 ± 0.09 | 317 | 15.1 ± 1.07 |
| *rab19* | 0.05 ± 0.05 | 4.86 ± 0.08 | 9.95 ± 0.11 | 309 | 14.7 ± 1.51 |
| *rab40* | 0.43 ± 0.11 | 5.71 ± 0.14 | 9.9 ± 0.14 | 131 | 6.24 ± 0.67 |
| *rabX1* | 0.7 ± 0.22 | 5.35 ± 0.13 | 9.6 ± 0.15 | 36 | 1.8 ± 0.24 |
| *rabX4* | 0.88 ± 0.18 | 7 | 13 | 1 | 0.05 ± 0.05 |

| **29 degree** | days until… (after egg collection) | | | # number of adults in total | Ø number of adults per vial |
| --- | --- | --- | --- | --- | --- |
|  | first instar larva | pupa | adult |  |  |
| control | 0 | 4.19 ± 0.09 | 7.95 ± 0.05 | 265 | 12.62 ± 1.5 |
| *rab4* | 0 | 4.1 ± 0.07 | 7.81 ± 0.09 | 255 | 12.14 ± 0.95 |
| *rab18* | 0.05 ± 0.05 | 4 | 6.86 ± 0.1 | 332 | 15.81 ± 1.86 |
| *rab19* | 0 | 4.05 ± 0.05 | 8.48 ± 0.11 | 194 | 9.24 ± 1.32 |
| *rabX1* | 0.5 ± 0.11 | 5.13 ± 0.17 | 8.9 ± 0.28 | 30 | 2.31 ± 0.36 |
| *rabX4* | 1.07 ± 0.07 | 7 ± 0.58 | 13 | 1 | 0.05 ± 0.05 |

**C: Validation of mutants over deficiencies**

| **18 degree** | days until… (after egg collection) | | | number of adults in total | Ø number of adults per vial |
| --- | --- | --- | --- | --- | --- |
|  | first instar larva | pupa | adult |  |  |
| control | 0.05 ± 0.05 | 8.5 ± 0.1 | 16.7 ± 0.14 | 290 | 13.81 ± 1.25 |
| *rab3*/*rab3* Df | 0.29 ± 0.1 | 8.52 ± 0.15 | 16.24 ± 0.17 | 96 | 4.57 ± 0.8 |
| *rab4*/*rab4* Df | 0.24 ± 0.1 | 9.19 ± 0.16 | 16.9 ± 0.23 | 233 | 11.1 ± 0.89 |
| *rab9*/*rab9* Df | 0.19 ± 0.09 | 7.57 ± 0.11 | 15.24 ± 0.15 | 168 | 8 ± 0.48 |
| *rab14*/*rab14* Df | 0.1 ± 0.07 | 7.62 ± 0.11 | 15.48 ± 0.15 | 192 | 9.14 ± 1.04 |
| *rab19*/*rab19* Df | 0.48 ± 0.11 | 8.24 ± 0.1 | 17.71 ± 0.3 | 142 | 6.76 ± 0.74 |
| *rab32*/*rab32* Df | 0.1 ± 0.07 | 9.67 ± 0.17 | 17.48 ± 0.13 | 192 | 9.14 ± 0.57 |
| *rab39*/*rab39* Df | 0.38 ± 0.13 | 8.62 ± 0.15 | 16.05 ± 0.11 | 124 | 5.9 ± 0.65 |
| *rab40*/*rab40* Df | 0.19 ± 0.09 | 9.9 ± 0.17 | 17.25 ± 0.3 | 116 | 5.52 ± 0.72 |
| *rabX1*/*rabX1* Df | 1.61 ± 0.14 | 9.67 ± 0.2 | 17.37 ± 0.35 | 85 | 4.25 ± 0.6 |
| *rabX4*/*rabX4* Df | 1.1 ± 0.18 | 11.11 ± 0.34 | 20.26 ± 0.49 | 57 | 2.85 ± 0.51 |
| *rabX6*/*rabX6* Df | 0.29 ± 0.1 | 9.45 ± 0.18 | 17.65 ± 0.13 | 153 | 7.65 ± 0.95 |

­

| **25 degree** | days until… (after egg collection) | | | number of adults in total | Ø number of adults per vial |
| --- | --- | --- | --- | --- | --- |
|  | first instar larva | pupa | adult |  |  |
| control | 0.05 ± 0.05 | 5.24 ± 0.1 | 9.19 ± 0.09 | 317 | 15.1 ± 1.07 |
| *rab19*/*rab19* Df | 0.29 ± 0.1 | 5.24 ± 0.1 | 10.1 ± 0.12 | 151 | 7.19 ± 0.69 |
| *rab40*/*rab40* Df | 0.33 ± 0.11 | 5.67 ± 0.14 | 9.38 ± 0.11 | 110 | 5.24 ± 0.1 |
| *rabX1*/*rabX1* Df | 0.76 ± 0.17 | 5.48 ± 0.13 | 9.35 ± 0.11 | 65 | 3.1 ± 0.34 |
| *rabX4*/*rabX4* Df | 1.36 ± 0.2 | 7.38 ± 0.18 | 12 ± 0.2 | 23 | 3.29 ± 0.52 |

| **29 degree** | days until… (after egg collection) | | | # number of adults in total | Ø number of adults per vial |
| --- | --- | --- | --- | --- | --- |
|  | first instar larva | pupa | adult |  |  |
| control | 0 | 4.19 ± 0.09 | 7.95 ± 0.05 | 265 | 12.62 ± 1.5 |
| *rab4*/*rab4* Df | 0.2 ± 0.09 | 4.3 ± 0.11 | 7.7 ± 0.11 | 236 | 11.8 ± 0.78 |
| *rab19*/*rab19* Df | 0.14 ± 0.08 | 4.1 ± 0.1 | 8.7 ± 0.1 | 117 | 5.57 ± 0.88 |
| *rabX1*/*rabX1* Df | 0.84 ± 0.14 | 4.75 ± 0.11 | 8.69 ± 0.12 | 24 | 1.2 ± 0.22 |
| *rabX4*/*rabX4* Df | 1 ± 0.12 | 5.71 ± 0.13 | 10.36 ± 0.2 | 26 | 1.86 ± 0.23 |
