# Supplementary material for: Systematic functional analysis of rab GTPases reveals limits of neuronal robustness to environmental challenges in flies
Source: eLife. 2021 Mar 5;10:e59594. doi: 10.7554/eLife.59594 (PMC8016483; doi:10.7554/eLife.59594)
Supplement: Transparent reporting form [file elife-59594-transrepform.docx]

***eLife’s* transparent reporting form**

We encourage authors to provide detailed information *within their submission* to facilitate the interpretation and replication of experiments. Authors can upload supporting documentation to indicate the use of appropriate reporting guidelines for health-related research (see [EQUATOR Network](http://www.equator-network.org/%20)), life science research (see the [BioSharing Information Resource](https://biosharing.org/)), or the [ARRIVE guidelines](http://www.plosbiology.org/article/info:doi/10.1371/journal.pbio.1000412) for reporting work involving animal research. Where applicable, authors should refer to any relevant reporting standards documents in this form.

If you have any questions, please consult our Journal Policies and/or contact us: [editorial@elifesciences.org](mailto:editorial@elifesciences.org).

**Sample-size estimation**

- You should state whether an appropriate sample size was computed when the study was being designed
- You should state the statistical method of sample size computation and any required assumptions
- If no explicit power analysis was used, you should describe how you decided what sample (replicate) size (number) to use

Please outline where this information can be found within the submission (e.g., sections or figure legends), or explain why this information doesn’t apply to your submission:

No specific power analyses were performed; sample sizes were chosen based on established protocols and statistical analyses for significance, as detailed for all experiments below.

**Replicates**

- You should report how often each experiment was performed
- You should include a definition of biological versus technical replication
- The data obtained should be provided and sufficient information should be provided to indicate the number of independent biological and/or technical replicates
- If you encountered any outliers, you should describe how these were handled
- Criteria for exclusion/inclusion of data should be clearly stated
- High-throughput sequence data should be uploaded before submission, with a private link for reviewers provided (these are available from both GEO and ArrayExpress)

Please outline where this information can be found within the submission (e.g., sections or figure legends), or explain why this information doesn’t apply to your submission:

The number of samples tested/analyzed is specified in the figure legends (Figure 1B; Figure 2M-N; Figure 3; Figure 4; Figure 5; Figure 6B-K, M-P; Figure 7A-D, F-S; Figure 1- figure supplement 2B; Figure 1- figure supplement 3B; Figure 2- figure supplement 2; Figure 4- figure supplement 1; Figure 5- figure supplement 1; Figure 6- figure supplement 1; Figure 7- figure supplement 1) or in the Material and Methods section (Figure 2; Figure 3; Figure 4D-F; Figure 6A & L; Figure 2- figure supplement 1). We did not perform any technical replications. Each sample was only tested/analyzed once. Outliers are included in the analysis and were not handled in any distinct way.

We did not have any special criteria for the exclusion/inclusion of data. No high-throughput sequence data was obtained during our study.

**Statistical reporting**

- Statistical analysis methods should be described and justified
- Raw data should be presented in figures whenever informative to do so (typically when N per group is less than 10)
- For each experiment, you should identify the statistical tests used, exact values of N, definitions of center, methods of multiple test correction, and dispersion and precision measures (e.g., mean, median, SD, SEM, confidence intervals; and, for the major substantive results, a measure of effect size (e.g., Pearson's r, Cohen's d)
- Report exact p-values wherever possible alongside the summary statistics and 95% confidence intervals. These should be reported for all key questions and not only when the p-value is less than 0.05.

Please outline where this information can be found within the submission (e.g., sections or figure legends), or explain why this information doesn’t apply to your submission:

We tested for normality in pilot experiments and chose the tests accordingly. All applied statistical analysis methods are listed in the respective figure legends (Figure 2A-L: unpaired, non-parametric Kolmogorov-Smirnov; Figure 2M-N: ordinary one-way ANOVA with pair-wise comparison; Figure 3A-B: ordinary one-way ANOVA with pair-wise comparison; Figure 3C-H: ordinary one-way ANOVA with group-wise comparison; Figure 4D-F: ordinary one-way ANOVA with pair-wise comparison; Figure 7C: ordinary one-way ANOVA with pair-wise comparison; Figure 2-figure supplement 1: unpaired, non-parametric Kolmogorov-Smirnov; Figure 2-figure supplement 2: ordinary one-way ANOVA with pair-wise comparison).

In the figures, mean with SEM (Figure 2A-L; Figure 3A-B; Figure 7C & E; Figure 2-figure supplement 1; Supplementary file 2), mean with SD (Figure 3C-H; Figure 4D-F; Figure 6A) and median (Figure 2M-N; Figure 2-figure supplement 2) are shown.

(For large datasets, or papers with a very large number of statistical tests, you may upload a single table file with tests, Ns, etc., with reference to sections in the manuscript.)

**Group allocation**

- Indicate how samples were allocated into experimental groups (in the case of clinical studies, please specify allocation to treatment method); if randomization was used, please also state if restricted randomization was applied
- Indicate if masking was used during group allocation, data collection and/or data analysis

Please outline where this information can be found within the submission (e.g., sections or figure legends), or explain why this information doesn’t apply to your submission:

No group allocation of the samples was used.

**Additional data files (“source data”)**

- We encourage you to upload relevant additional data files, such as numerical data that are represented as a graph in a figure, or as a summary table
- Where provided, these should be in the most useful format, and they can be uploaded as “Source data” files linked to a main figure or table
- Include model definition files including the full list of parameters used
- Include code used for data analysis (e.g., R, MatLab)
- Avoid stating that data files are “available upon request”

Please indicate the figures or tables for which source data files have been provided:

No source data files haven been submitted.
